# A Comprehensive HPLC-HRMS/MS Targeted Screening Method to Detect 90 New Psychoactive Substances in Oral Fluid Samples

**DOI:** 10.3390/biology15080616

**Published:** 2026-04-13

**Authors:** Ilaria Spinella, Fabio Altieri, Simona Pichini, Adele Minutillo, Annagiulia Di Trana

**Affiliations:** 1 Department of Biochemical Sciences “Rossi Fanelli”, “La Sapienza” University of Rome, Piazzale Aldo Moro 5, 00185 Rome, Italy; spinella.1902876@studenti.uniroma1.it (I.S.); fabio.altieri@uniroma1.it (F.A.); 2 National Center on Addiction and Doping, National Institute of Health, Viale Regina Elena 299, 00161 Rome, Italy; simona.pichini@iss.it (S.P.); adele.minutillo@iss.it (A.M.)

**Keywords:** New Psychoactive Substances, screening method, HPLC-MS/HRMS, synthetic cathinones, nitazenes, synthetic cannabinoids, oral fluid

## Abstract

The continuous emergence of New Psychoactive Substances (NPS) poses a significant challenge to public health and forensic toxicology due to their unpredictable pharmacology and rapid turnover on the illicit market. This study describes the development and validation of a high-resolution screening method for the simultaneous detection of 90 NPS in oral fluid (OF), a matrix of choice for non-invasive sampling and roadside testing. The analytical workflow utilizes a “dilute-and-shoot” approach (1:2 *v*/*v* dilution) followed by ultra-high-performance liquid chromatography coupled with a quadrupole-Orbitrap hybrid mass spectrometer (UHPLC-HRMS/MS). Chromatographic separation was achieved in 11 min using a biphenyl column and a gradient elution. The method was validated according to ANSI/ASB Standard 036 guidelines, covering 90 substances including synthetic cannabinoids (e.g., HHC, MDMB-4en-PINACA), synthetic cathinones, and high-risk synthetic opioids such as nitazenes and fentanyl analogues. Results showed high sensitivity, with limits of identification (LOI) reaching 1 ng/mL for 44.4% of the analytes and 5 ng/mL for 37.8%, while the remaining compounds showed higher LOIs ranging from 10 to 100 ng/mL. No significant matrix interference or carryover was observed. The method was successfully applied to real samples from external quality control programs and forensic cases. This robust and versatile screening tool is suitable for clinical and forensic applications, supporting the monitoring of emerging NPS trends.

## Simple Summary

New Psychoactive Substances are a vast group of over 1000 drugs with diverse psychotropic effects. Due to their rapid emergence on the illegal market, they often linger in a legal “grey area”. Their prompt identification could be of vital importance not only to confirm consumption but also to assess impairment in cases of intoxication or “drugged driving”. Unfortunately, most current analytical methods usually cover just a few of these substances, often missing the newest classes entirely. To fill this gap, we developed a new method capable of identifying 90 New Psychoactive Substances simultaneously using saliva as a matrix. The method also included a panel of the most recent molecules. The assay was successfully validated according to the most recent international guidelines, and tested on 21 real saliva samples. It correctly identified the contained substance in 88% of cases, with only 1 false positive and 1 false negative. These results prove that our method is both effective and efficient, making it a powerful tool for emergency rooms, roadside testing, and forensic investigations.

## 1. Introduction

New Psychoactive Substances (NPSs) are substances of abuse that are not controlled under the 1961 Single Convention on Narcotic Drugs and the 1971 Convention on Psychotropic Substances, as officially defined by the United Nations Office on Drugs and Crime (UNODC) [1]. This definition encompasses a broad range of substances, divided into different pharmacological classes such as synthetic cannabinoids (SCRAs), synthetic cathinones (SC), synthetic opioids, natural NPSs, hallucinogens, designer benzodiazepines, phenethylamines and stimulant NPS. These molecules have increasingly flooded onto illicit markets due to their psychotropic properties and their controversial legal status, representing an alternative to classic drugs of abuse. The most recent data reports more than 500 different NPSs identified in the global NPS market, predominantly SCRAs (34% in 2023) and SCs (23% in 2023) [2].

According to the UNODC, the spread of NPSs has globally expanded, becoming a major problem in Central Asia and Eastern Europe [3]. SCRAs are currently the most numerous class on the global market, with 130 analogues, followed by synthetic stimulants, particularly SCs (106) and phenethylamines (84) [4]. In Europe, the most represented classes by number of analogues are SCRAs (73), SCs (64), and phenethylamines (39) [5]. Although synthetic opioids are fewer in number (19 analogues), they pose a serious public health threat [5]. These data highlight that, despite some stabilisation in the total number of new substances, NPSs with high toxicological risk continue to emerge, underscoring the need for sensitive and comprehensive analytical methods. Seizures of high-risk subclasses, such as nitazenes, are also increasing [2].

However, the number of NPSs and the prevalence of the different classes fluctuate regionally every year, with irregular trends. Furthermore, the unpredictable pharmacology of the emerging NPSs often leads to unforeseen effects, which can cause lethal intoxications [6,7,8,9]. This phenomenon represents an increasing challenge for public health, raising concerns for international agencies [3]. Therefore, the analytical detection and identification of NPSs in biological matrices are crucial in forensic and clinical toxicological analyses to confirm their consumption and the possible implications in intoxication cases. Whereas the difficulties related to the analytical confirmation of new molecules, requiring advanced instrumentation, such as NMR, to clarify the molecular structure, other aspects further complicate NPS analyses in biological matrices. The constant emergence of new analogues, often related to SCRAs, SCs, and opioids, together with the presence of numerous structural isomers, makes their analytical identification particularly challenging [3]. The number of isomers with strictly related structures could not be resolved through common immuno-enzymatic analytical methods. Furthermore, the chromatographic co-elution of isobaric compounds is hardly resolved by common mass spectrometers [10]. Another important aspect is related to the pattern of consumption of the NPSs, which are often inadvertently consumed in place of other drugs or as adulterants of the main drug of abuse, resulting in very low concentrations in the biological matrices and/or multiple drug consumption [11,12].

As a result, there is still a lack of comprehensive screening methods capable of simultaneously identifying a broad range of NPS in complex biological matrices with high specificity and sensitivity. Indeed, the multi-analytical approach was adopted for the broad screening of OF samples from driving under the influence of drugs in Belgium to expand the panel of detected substance, including the LC-MS/MS method and the LC-HRMS method [12]. In this context, oral fluid (OF) represents a valuable matrix, as it reflects recent drug intake, correlates with blood concentrations, and can be collected rapidly and non-invasively. Moreover, OF is increasingly recognized as a matrix of choice in toxicological screening, since several studies have demonstrated a correlation between OF drug concentrations and the occurrence and intensity of psychotropic effects [13,14]. Recently, the LC-HRMS method was applied to retrospectively analyse OF samples from psychiatric patients to assess NPS consumption in Sweden [15]. Although the method included a high number of NPSs, some important classes such as nitazenes or orphines were not mentioned, affecting its applicability in other context.

The present study describes the development and validation of a UHPLC-HRMS/MS method for the simultaneous screening of multiple classes of NPS in OF. The relevance of OF analysis is further supported by recent regulatory developments in Italy, where this matrix has been officially adopted for roadside toxicological testing [16]. The combination of OF sampling with high-resolution mass spectrometry (HRMS) provides a robust and versatile analytical approach for clinical, forensic, and road safety applications. While several LC-MS-based methods for the detection of NPS in OF have been reported in the literature, many of them are focused on single classes of substances or require labor-intensive sample preparation procedures [17]. These limitations reduce their flexibility and applicability in high-throughput screening contexts, particularly in forensic and public health settings. The aim of the present study was to develop and validate a qualitative UHPLC-HRMS/MS screening method for the simultaneous detection of a wide panel of NPS in OF, including recently emerged compounds of toxicological relevance. The method was designed to combine broad analytical coverage with high specificity, while minimizing sample preparation through a simple “dilute and shoot” approach [18]. This workflow provides a practical and reliable tool for clinical, forensic, and road safety applications [6].

## 2. Materials and Methods

### 2.1. Chemicals and Reagents

The list of methanolic standard solutions of target analytes is reported in Table 1, along with their concentrations and suppliers. UHPLC-grade methanol (MeOH), ultrapure water, ammonium formiate (10 mM), and analytical grade formic acid were supplied by Sigma-Aldrich (Milan, Italy). The calibrant solution used for mass spectrometer calibration was supplied by Thermo Fisher Scientific (Waltham, MA, USA).

### 2.2. Standard Solutions and Biological Samples

Individual standard solutions were prepared in methanol (MeOH):acetonitrile (ACN) (50:50, *v*/*v*) at a concentration of 1 μg/mL for spectrometric characterization, while retention times were determined by preparing standard solutions at 1 μg/mL in mobile phase A (0.1% formic acid + 2 mM ammonium formate, MPA):mobile phase B (0.1% formic acid in ACN:MeOH (50:50, *v*/*v*) + 2 mM ammonium formate, MPB) (80:20, *v*/*v*). Based on the retention times, all the analytes were grouped into 8 Working Groups (WGs), and the corresponding methanolic solutions were prepared by mixing the stock standard solution and a proper volume of MeOH to obtain 8 solutions at different concentrations.

The method was validated in OF using WG solutions prepared at different concentrations. For each WG, the analytes were mixed in MeOH and vortexed to ensure complete homogenization. Final WG stock solutions were prepared at concentrations of 1 μg/mL, 200 ng/mL, 100 ng/mL, 50 ng/mL, and 10 ng/mL. For validation experiments, WG solutions were diluted in OF following a “dilute and shoot” approach using a mobile phase mixture of MPA:MPB (80:20, *v*/*v*), yielding final concentrations in OF of 100 ng/mL, 20 ng/mL, 10 ng/mL, 5 ng/mL, and 1 ng/mL. After dilution, samples were vortexed, centrifuged, and transferred to vials equipped with internal inserts prior to instrumental analysis.

### 2.3. Oral Fluid Samples Preparation and Real Samples

Pretested blank OF samples were obtained from 10 healthy donors, not exposed to drugs of abuse, to assess any interference and prepare fortified samples. The absence of psychoactive substances in the matrix was verified using a routine analytical method. The blank samples were stored at −20 °C until analysis. For the method validation, blank samples were fortified at different concentrations with the analytes methanolic solutions. Real OF samples from previous studies and the inter-laboratory external quality control program (ORALVEQ) were also analyzed. The real samples were subjected to the same preparation procedures as the fortified samples and analyzed by UHPLC-HRMS/MS.

OF samples were prepared at four different dilution ratios (1:2, 1:3, 1:5, 1:10 *v*/*v*) and used for the steps described in the following paragraphs. Each sample was prepared by adding 10 μL of WG to 100 μL OF, vortexed, diluted with 100, 200, 400 or 900 μL MPA:MPB, depending on the dilution ratio considered, then vortexed again, centrifuged for 6 min at 5000 rpm, and transferred to vials with internal inserts for instrumental analysis. Having identified the 1:2 *v*/*v* dilution as optimal, all subsequent analyses were conducted using this dilution.

Eight real OF samples stored at −80 °C in the Istituto Superiore di Sanità storage room for research purposes and 13 samples from the inter-laboratory quality assurance program (ORALVEQ) [19] were analyzed. All the samples were collected according to a previously approved protocol which was approved by the local Ethical Committee for human research (CEI-HUGTiP ref. PI-18-267). A volume of 100 μL of each sample was collected and diluted with 100 μL of MPA:MPB mobile phase mixture (80:20, *v*/*v*) according to the 1:2 dilution ratio deemed optimal during validation. The final solutions were vortexed, centrifuged for 6 min at 5000 rpm, and transferred to vials with internal inserts for instrumental analysis. All real samples were analyzed using the same chromatographic and spectrometric conditions.

### 2.4. Instrumental Conditions

Chromatographic separation was carried out with a DIONEX Ultimate 3000 HPLC/UHPLC liquid chromatography system equipped with a Force Inert Biphenyl (3 μm, 50 × 3.0 mm) from Restek s.r.l. (Milan, Italy), set at 40 °C during the analysis. A gradient elution was performed using two mobile phases: MPA consisting of an aqueous solution with 2 mM ammonium formate and 0.1% formic acid (*v*/*v*), and MPB composed of ACN and MeOH (50:50, *v*/*v*) containing 2 mM ammonium formate and 0.1% formic acid (*v*/*v*). The chromatographic separation was performed using a gradient elution of MPA and MPB at a flow rate of 0.4 mL/min. The initial conditions were set at 99:1 (MPA:MPB) and were held for 1 min. Subsequently, from 1 to 9 min, MPA was decreased from 99% to 1% and MPB was increased from 1% to 99%. This composition was maintained for 3.5 min, after which the system was rapidly returned to the initial conditions (99:1, MPA:MPB), and re-equilibrated for 2.99 min. The injection volume was 10 μL for all samples. Mass spectrometric analysis was performed on a Q Exactive^TM^ Focus quadrupole-Orbitrap^TM^ hybrid mass spectrometer from Thermo Fisher Scientific (Waltham, MA, USA) equipped with a heated electrospray ionization (HESI) source working in positive ionization mode. The spray voltage was set at 3.90 kV, sheath gas flow at 25, auxiliary gas flow of 15, capillary temperature at 320 °C, auxiliary gas heater temperature of 0, and S-lens RF level of 50. The mass spectrometric acquisition was performed in Full Mass (Full MS)/data-dependent MS/MS (ddMS^2^) acquisition mode. Full MS spectra were acquired over an *m*/*z* range of 50–650 at a resolution of 70,000 with an automatic gain control (AGC) target of 1 × 10^5^ and automatic maximum injection time (IT), while ddMS^2^ acquisition was performed at a resolution of 17,500 using a 1.0 *m*/*z* isolation window and stepped normalized collision energies (NCE) of 20, 35, and 65, with 2 × 10^4^ ACG target automatic maximum IT. An inclusion list containing target ions, *m*/*z* values, and retention times was applied to optimise precursor selection. The instrument was calibrated in positive ionization mode prior to analysis using a certified calibrant solution. Compound identification was performed using a mass accuracy window of ±5 ppm and retention time tolerance of 0.02 min.

### 2.5. Method Validation

The method was fully validated following a 5-day validation protocol, in accordance with the most recent guidelines for method validation in analytical toxicology, ANSI/ASB Standard 036 (2019) [20], and following the specific recommendations for method validation of HRMS screening methods [21].

Matrix interference, defined as any signal arising from the sample matrix that could interfere with the identification of the target analytes, was evaluated by analyzing ten blank OF samples at dilution ratios of 1:2, 1:5, and 1:10, monitoring for peaks at the retention times of the analytes. Method specificity, defined as the ability to distinguish the target analytes from other components in the sample, including co-eluting compounds, was evaluated by extracting the masses of target analytes belonging to all WSGs not present in the samples. Method sensitivity, defined as the Limit of Identification (LOI), represents the lowest analyte concentration that can be reliably detected. As defined by Wille et al. [21], LOI differs from the limit of detection (LOD) as it requires reliable compound identification rather than simple detection. A substance was considered detectable if both the chromatographic peak related to the extraction of the precursor ion and the fragmentation spectrum of the molecule were present at the retention time observed during the characterization. The criteria and the corresponding information for each analyte are reported in Table S1 (Supplementary Materials). LOI was determined using OF samples fortified at decreasing concentrations (20, 10, 5, and 1 ng/mL) and analyzed at dilution ratios of 1:2, 1:3, and 1:5. Carryover, defined as the residual signal from a previously analyzed high-concentration sample appearing in subsequent blank samples, was assessed by injecting blank samples immediately after samples fortified at 100 ng/mL; carryover was considered negligible when no signal was observed. All measurements were performed in triplicate [6].

## 3. Results

### 3.1. Method Development

The progressive optimization of chromatographic and spectrometric conditions allowed us to set up a sensitive UHPLC-HRMS/MS screening method for the reliable identification of 90 NPSs in OF. Injecting the individual analyte solution directly into the HESI source, the hydrogen adduct [M+H]^+^ was consistently selected as the best precursor ion in positive mode for all the analytes. The single [M+H]^+^ adducts were subsequently fragmented at increasing NCE to assess the most appropriate collision energies to obtain the most informative fragmentation patterns for all compounds. Therefore, the selected stepped energies at 20, 35, and 65 NCE represented the best compromise. Afterwards, the chromatographic conditions were optimized by comparing different chromatographic columns, mobile phase compositions, and elution gradients to achieve satisfactory chromatographic resolution for all analytes. Representative chromatograms and mass spectra of the precursor ions and major fragments for all analytes were obtained during method characterization under the final analytical conditions, as reported in Section 2. Finally, the analytical conditions were tested in spiked OF samples to confirm the analytes’ detectability and Rt, which were finally noted in the inclusion list to obtain single acquisition windows and increase the spectrometer capacity. Figures S1–S8 show all standards of WG1 elute between 0 and 4.65 min, WG2 between 4.65 and 5.10 min, WG3 between 5.10 and 5.60 min, WG4 between 5.60 and 6.10 min, WG5 between 6.10 and 6.70 min, WG6 between 6.70 and 8.50 min, WG7 between 8.10 and 9.10 min, and WG8 between 9.10 and 11 min. Figures S1–S8 present the chromatograms and spectra of the analytes divided by WGs for illustrative purposes, reflecting the retention time ranges used to assign the compounds to the respective WGs.

Sample preparation was optimized by spiking analyte solutions into OF at 100 ng/mL and diluting with MPA:MPB (80:20 *v*/*v*). Three dilution ratios (1:2, 1:3, and 1:5 *v*/*v*) were evaluated, with 1:2 *v*/*v* providing the best peak shape and being selected as the optimal condition.

### 3.2. Method Validation

**Figure 1:**
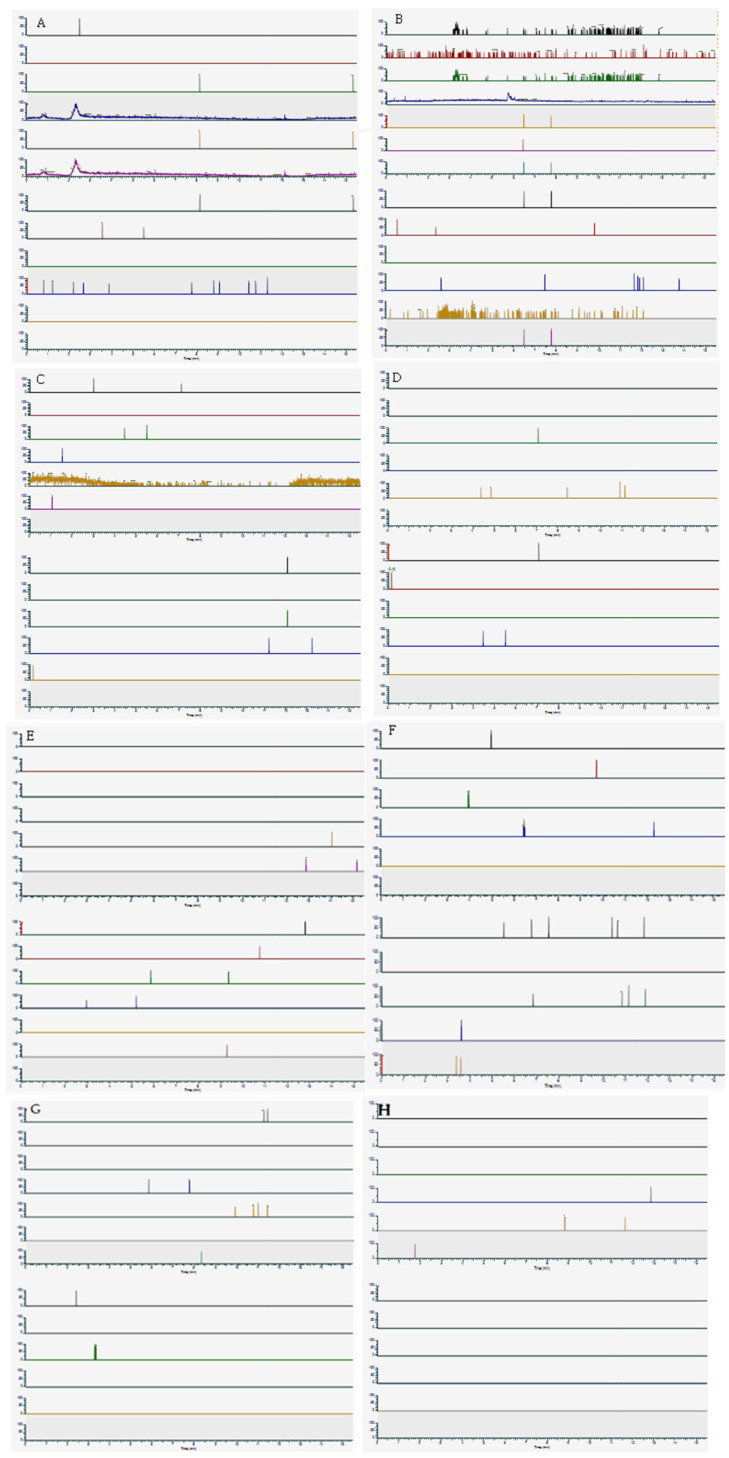
Extraction of analytes for matrix interference evaluation. (**A**) WG1; (**B**) WG2; (**C**) WG3; (**D**) WG4; (**E**) WG5; (**F**) WG6; (**G**) WG7; (**H**) WG8.

Matrix interference was studied by analysing ten blank OF samples, prepared under the same conditions as the real samples. In particular, the extracted chromatogram for each [M+H]^+^ adduct was evaluated for the eventual presence of confounding chromatographic peaks, which were not observed at any Rt, as shown in Figure 1.

The sensitivity of the method was determined by analyzing OF samples fortified at decreasing concentrations of 20 ng/mL, 10 ng/mL, 5 ng/mL, and 1 ng/mL. Sensitivity was evaluated at a 1:2, 1:3, and 1:10 dilution ratios, but the results were considered using the optimal ratio 1:2. Substance detectability was considered in the presence of both the chromatographic peak of the precursor ion and the fragmentation spectrum of the molecule at the same retention time observed during characterization. All analytes that did not meet both conditions were considered non-detectable. Furthermore, S/N = ∞ was observed for the majority of analytes. The lowest concentration at which the minimum analyte recognition conditions occurred was considered the Limit of Identification (LOI) of the method. At a 1:2 dilution ratio, 44.4% of the analytes reached the LOI at 1 ng/mL, 37.8% at 5 ng/mL, 7.8% at 10 ng/mL, 4.4% at 20 ng/mL, and 5.6% at 100 ng/mL (Table 2).

The method specificity was evaluated on the spiked OF samples at all the concentrations without observing any inter-analyte interferences. Carryover was assessed by monitoring the presence of analytical residues in blank samples injected after OF samples fortified at high concentrations (100 ng/mL), in at least three replicates. All the relevant parameters are reported in Table 2.

### 3.3. Real OF Samples

**Figure 2:**
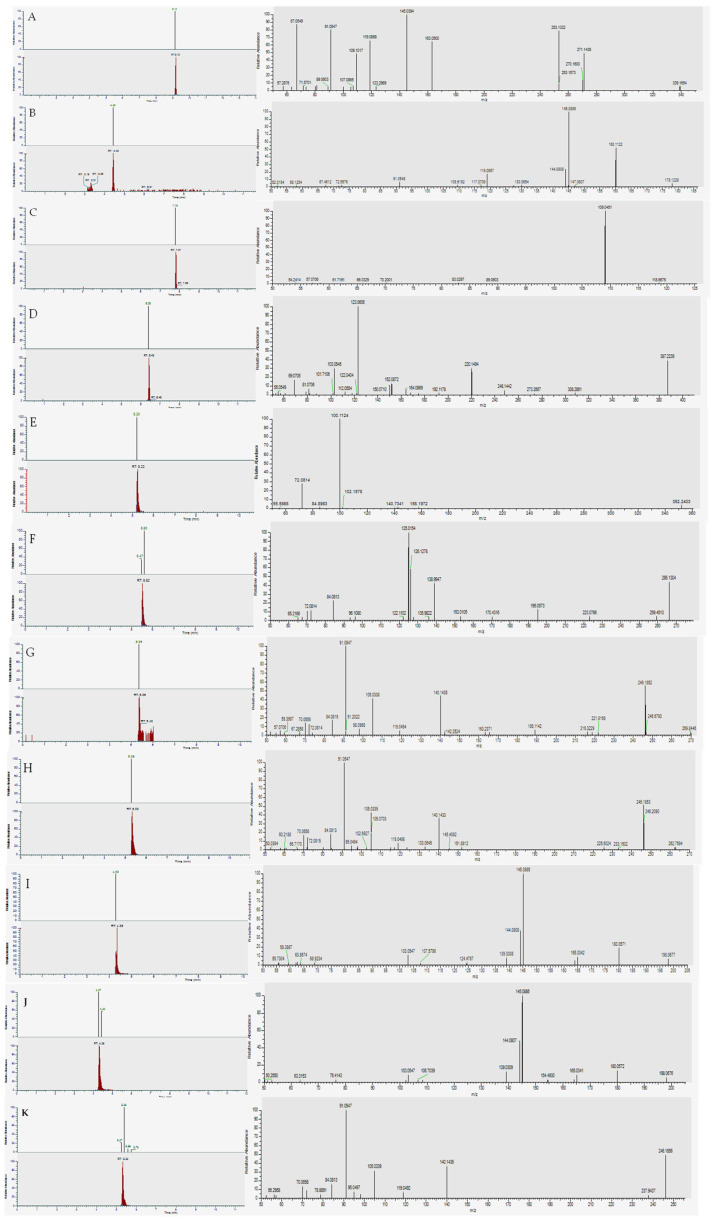
Analysis of real OF samples. (**A**) OF3; (**B**) OF4; (**C**) OF5; (**D**) OF7; (**E**) OF9; (**F**) OF11; (**G**) OF13; (**H**) OF18; (**I**) OF19; (**J**) OF19; (**K**) OF21.

The validated method was applied to the analysis of 21 real OF samples, proving its applicability in the routine. The five blank samples were correctly individuated, while 5 more samples tested negative due to the presence of non-validated substances. One sample (OF21) resulted as a false negative, while in one sample containing two NPS (OF18), only one NPS was detected, resulting in a partial positive. Only one false positive was observed. Overall, 19 out of 21 samples (90.5%) were correctly classified, demonstrating the method’s effectiveness in real sample analysis. The detailed results of the analyzed samples are reported in Table 3, while chromatograms and fragmentation spectra from the positive samples are shown in Figure 2. All samples were analyzed on the same day to ensure consistent experimental conditions. To verify reproducibility, each sample was reanalyzed on three consecutive days, consistently yielding identical results to those obtained in the initial analytical batches, confirming the reliability and robustness of the method.

## 4. Discussion

A comprehensive screening method using UHPLC-HRMS/MS to detect 90 NPSs in OF has been successfully validated, allowing its application to 21 pretested real samples. The method was designed to detect a broad panel of NPSs, including analogs of the most recent emerging classes such as “orphines” and last generation SCs. In particular, the non-fentanyl synthetic opioids have been replacing the fentanyl analogues on the illicit market since 2020, with a constant increase in popularity among users, which is currently alerting the international agencies and governments to a new wave of opioid crises [22,23]. In this regard, the present method is the first multi-class screening method allowing the detection of brorphine in OF. α-PHiP [24] and MDPHP [25] were also included due to their increasing prevalence on the Italian illicit market and their association with severe psychostimulant effects and intoxications, as reported in the literature.

OF was selected as the target matrix due to its non-invasive collection, rapid sampling, and ability to provide reliable information on recent drug intake. Its use is also supported by Italian regulations, which have officially adopted OF for roadside toxicological testing [16]. Moreover, OF analyses have already proved to be suitable for drug detection in clinical contexts or epidemiological studies on NPS [12,15]. A key advantage of the proposed method is its ability to detect multiple chemical classes simultaneously with a simple “dilute and shoot” approach [18]. This reduces sample handling, saves time, and allows high-throughput screening in clinical and forensic laboratories. Compared with more labor-intensive extraction techniques reported in the literature, such as microextraction on packed sorbent (MEPS) [26], liquid–liquid extraction [27], or parallel artificial liquid membrane extraction (PALME) [28], this approach provides several practical benefits. These techniques usually involve several preparation steps, longer processing times, and sometimes higher solvent consumption or specific optimization for certain compounds. In contrast, the dilute and shoot approach reduces sample handling, shortens preparation time, lowers the risk of analyte loss, and requires smaller volumes of OF [27]. It also allows high-throughput analysis while maintaining good sensitivity and reproducibility. The dilute and shoot approach for NPS detection in OF was applied by Malaca et al. in a quantitative LC-MS/MS assay to detect 13 classic drugs of abuse and NPSs. Despite the limited number of substances, the method showed satisfactory validation parameters [18]. An expanded panel of NPSs was included in a validated assay to detect 77 NPSs and 24 classic drugs of abuse in OF through LC-MS/MS, which used the dilute and shoot approach with M3^®^ solvent (Comedical, Trento, Italy) [17]. In this case, the M3^®^ buffer stabilized the analytes, increasing the cost of analysis. Furthermore, the absence of interferences from the matrix proved that the approach could sufficiently prepare the samples for the instrumental analysis. The use of HRMS combined with chromatographic separation provides high analytical specificity. HRMS allows the discrimination of structurally related compounds, including positional isomers and isobaric species, which are common among NPSs [17]. We obtained the separation of structural isomers, such as α-PHP and α-PHiP, positional isomers such as 2-CMC and 3-CMC, *cis* 3-methyl norfentanyl and *trans* 3-methyl norfentanyl as shown in Supplementary Materials. Accurate mass measurements, isotopic pattern analysis, and informative MS/MS fragmentation spectra all contribute to confident identification, bypassing the analogues’ co-elution-related issues. Moreover, the combination of Full MS and ddMS^2^ acquisition allows both untargeted screening and confirmatory analysis in a single workflow, enhancing overall robustness. Sensitivity was another critical factor in method development. Method sensitivity was sufficient to reliably detect 40 out of 90 target analytes at 1 ng/mL, while all compounds were detectable at higher concentrations (ranged from 5 to 100 ng/mL), reflecting uneven performance across different chemical classes. In general, substances characterized by higher LOI values, such as SCRAs, are typically consumed at higher doses, partially compensating for the reduced analytical sensitivity, which remains adequate for practical screening since typical NPS concentrations in OF are generally higher than the method’s LOI for most substances [29]. The reduced sensitivity for such substances could be attributed to the poor ionization efficiency at the applied source conditions, which represents the best compromise to detect the extensive panel included in the method. Nonetheless, caution is warranted when interpreting negative results for these compounds or in cases of low-dose exposure. In contrast, opioids showed low LOI values (1 ng/mL) for most of the compounds, reflecting their high pharmacological potency. The optimal dilution factor of 1:2 was selected after testing several ratios (1:2, 1:3, 1:5, and 1:10) to balance matrix effects and analyte detectability. Higher dilutions reduced matrix interference but decreased signal intensity, while lower dilutions increased background noise. This finding emphasizes the importance of carefully optimizing sample preparation for qualitative screening. Carryover tests confirmed that residual analytes did not affect subsequent injections, supporting the reliability of the method for analyzing high-concentration samples. Application to real OF samples confirmed the robustness and practical applicability of the method. The procedure reliably detected multiple NPSs in samples with varying composition, providing correct results in 90.5% of the cases. Out of the 21 samples, 10 were negative, with five containing no substances and five positives for compounds not included in the panel, the partial detection of a single analyte, and one false positive. The reliability rate of the method was 93%, matching the proposed acceptance criteria of Lemyre et al. [30]. These rare discrepancies could be attributed to degradation of the analytes, in case of false negatives, or unexpected interference from the matrix due to rare endogenous analytes. Despite these limitations, NPSs from multiple chemical classes including SCRAs, SCs, and opioids were reliably detected, confirming the method’s effectiveness in real biological samples and its practical applicability in forensic and NPS monitoring contexts. Another important limitation is the coelution of some positional isomers, such as 3-MMC and 4-MMC and 3-CMC and 4-CMC, whose exact discrimination requires a specific method during confirmatory step analysis. Nonetheless, certain limitations should be acknowledged. As a qualitative screening method, the approach is not intended for accurate quantification, and differences in sensitivity were observed among compounds belonging to different chemical classes. Furthermore, the method’s performance depends on chromatographic and dilution parameters, which may require adjustment when incorporating new NPSs into the panel. Continuous updating of the target list will be necessary to maintain the relevance of the method in the rapidly evolving NPS market. For each new substance, retention time and characteristic fragmentation spectra must be determined, and the method must be partially revalidated to confirm reliable detection under the established analytical conditions [31]. The UHPLC-HRMS/MS method developed allows incorporation of emerging NPSs without changing the overall sample preparation or chromatographic setup, ensuring the method remains practical and applicable for forensic, clinical, and roadside monitoring.

## 5. Conclusions

The UHPLC-HRMS/MS method presented allows the simultaneous screening of 90 NPSs belonging to different chemical classes in OF, providing high analytical specificity and suitable sensitivity for routine toxicological applications. The use of HRMS enables the reliable identification of isobaric compounds and positional isomers, supporting the detection of structurally diverse and emerging NPSs.

The main strength of the method lies in the simple and rapid “dilute and shoot” sample pretreatment, requiring only 100 μL of OF and minimal handling, which makes it easily applicable in high-throughput clinical and forensic laboratories. Validation and application to real samples confirmed the robustness and practical applicability of the procedure. As for any screening approach, positive findings should be confirmed by dedicated confirmatory methods. Owing to its flexibility and short analysis time, the method can be readily expanded to include newly emerging NPSs and represents an effective tool for OF-based toxicological screening.

## Figures and Tables

**Table 1:** New Psychoactive Substances standard solution concentrations and corresponding suppliers, grouped per pharmacological class.

Substances	Supplier	Concentration
Arylcyclohexylamines		
2-fluorodeschloroketamine hydrochloride	Comedical s.r.l. (Trento, Italy)	20 μg/mL
3-hydroxyphencyclidine hydrochloride	Comedical s.r.l.	10 μg/mL
3-methoxeticyclidine hydrochloride	Comedical s.r.l.	20 μg/mL
Deschloro-N-etyl ketamine hydrochloride	Comedical s.r.l.	20 μg/mL
Designer benzodiazepines		
Fluetizolam	Comedical s.r.l.	10 μg/mL
Synthetic cannabinoids		
1CP-LSD	Comedical s.r.l.	10 μg/mL
4-fluoro MDMB-BUTINACA	Comedical s.r.l.	10 μg/mL
4-Fluoro MDMB-BUTICA	Comedical s.r.l.	10 μg/mL
5-chloro THJ 018	Comedical s.r.l.	50 μg/mL
5C-MDA-19	Comedical s.r.l.	10 μg/mL
5-fluoro NNEI 2′-napthyl isomer	Comedical s.r.l.	50 μg/mL
5-fluoro APP-PICA	Comedical s.r.l.	50 μg/mL
5-fluoro CUMYL Pegaclone	Comedical s.r.l.	20 μg/mL
5-fluoro CUMYL-P7AICA	Comedical s.r.l.	20 μg/mL
5-fluoro CUMYL-PICA	Comedical s.r.l.	10 μg/mL
5-fluoro CUMYL-PINACA	Comedical s.r.l.	50 μg/mL
5-fluoro MDMB-7-PAICA	Comedical s.r.l.	20 μg/mL
9-hexahydrocannabinol	Comedical s.r.l.	10 μg/mL
ADB-5Br-INACA	Comedical s.r.l.	10 μg/mL
ADB-4en-PINACA	Comedical s.r.l.	10 μg/mL
ADB-BUTINACA	Comedical s.r.l.	10 μg/mL
AM2233	Comedical s.r.l.	100 μg/mL
APP FUBINACA	Comedical s.r.l.	50 μg/mL
BZO-4en-POXIZID	Comedical s.r.l.	10 μg/mL
CUMYL-CH-MeGACLONE	Comedical s.r.l.	10 μg/mL
CUMYL-NBMINACA	Comedical s.r.l.	10 μg/mL
EDMB-PINACA	Comedical s.r.l.	10 μg/mL
JWH-147	Comedical s.r.l.	100 μg/mL
JWH-016	Comedical s.r.l.	100 μg/mL
MDMB-4en-PICA	Comedical s.r.l.	10 μg/mL
MDMB-4en-PINACA butanoic acid metabolite	Comedical s.r.l.	10 μg/mL
MDMB-BUTINACA	Cayman Chemical Company (Ann Arbor, MI, USA)	1 mg/100 μL
MMB2201	Comedical s.r.l.	50 μg/mL
Pravadoline	Comedical s.r.l.	100 μg/mL
Synthetic cathinones		
1-Naphyrone	Comedical s.r.l.	100 μg/mL
2-methyl-α-pyrrolidinopropiophenone	Comedical s.r.l.	10 μg/mL
3-chloromethcathinone hydrochloride	Comedical s.r.l.	10 μg/mL
3-methylmethcathinone hydrochloride	Comedical s.r.l.	10 μg/mL
3,4-trimethylene-α-Piperidinovalerophenone hydrochloride	Comedical s.r.l.	10 μg/mL
3-fluoro-α-pyrrolidinovalerophenone	Comedical s.r.l.	1 mg/mL
4-bromomethcathinone hydrochloride	Comedical s.r.l.	1 mg/mL
4-chloromethcathinone hydrochloride	Cayman Chemical Company	10 μg/mL
4-chloro-α-pyrrolidinovalerophenone hydrochloride	Comedical s.r.l.	10 μg/mL
4-ethylethcathinone hydrochloride	Comedical s.r.l.	1 mg/mL
4-fluoro-α-pyrrolidinohexanophenone hydrochloride	Comedical s.r.l.	1 mg/mL
4-methyl N ethyl cathinone metabolite hydrochloride	Comedical s.r.l.	1 mg/mL
4-methyl-α-pyrrolidinohexanophenone hydrochloride	Comedical s.r.l.	50 μg/mL
2-chloromethcathinone hydrochloride	Comedical s.r.l.	10 μg/mL
2-methylmethcathinone hydrochloride	Comedical s.r.l.	10 μg/mL
3,4-methylenedioxy-α-pyrrolidinohexanophenone hydrochloride	Comedical s.r.l.	10 μg/mL
4-fluoromethcathinone metabolite	Cayman Chemical Company	1 mg/mL
N-cyclohexyl butylone hydrochloride	Comedical s.r.l.	10 μg/mL
N-ethyl heptedrone hydrochloride	Comedical s.r.l.	10 μg/mL
N-ethyl pentylone hydrochloride	Comedical s.r.l.	20 μg/mL
α-pyrrolidinohexanophenone	Comedical s.r.l.	20 μg/mL
α-pyrrolidinohexanophenone metabolite hydrochloride	Cayman Chemical Company	1 mg/mL
α-pyrrolidinoisohexanophenone hydrochloride	Comedical s.r.l.	1 mg/mL
β-pentedrone hydrochloride	Comedical s.r.l.	100 μg/mL
Phenethylamines		
6-MAPB hydrochloride	Comedical s.r.l.	50 μg/mL
4-bromo-2,5-dimethoxyphenylethylamine	Comedical s.r.l.	100 μg/mL
Synthetic opioids		
Acetylfentanyl hydrochloride	Comedical s.r.l.	50 μg/mL
AP-237 hydrochloride	Comedical s.r.l.	20 μg/mL
AP-238 hydrochloride	Comedical s.r.l.	1 mg/mL
Brorphine hydrochloride	Comedical s.r.l.	10 μg/mL
Butyryl fentanyl hydrochloride	Comedical s.r.l.	50 μg/mL
Butyryl norfentanyl hydrochloride	Comedical s.r.l.	50 μg/mL
Butonitazene	Comedical s.r.l.	10 μg/mL
Cyclopropylfentanyl hydrochloride	Comedical s.r.l.	50 μg/mL
Cis-3-methyl norfentanyl	Comedical s.r.l.	50 μg/mL
Ethyleneoxynitazene citrate	Comedical s.r.l.	10 μg/mL
Etodesnitazene citrate	Comedical s.r.l.	10 μg/mL
Phenyl fentanyl hydrochloride	Comedical s.r.l.	50 μg/mL
Furanyl fentanyl	Comedical s.r.l.	20 μg/mL
Furanyl norfentanyl hydrochloride	Comedical s.r.l.	50 μg/mL
Metodesnitazene hydrochloride	Comedical s.r.l.	10 μg/mL
Methoxyacetyl fentanyl hydrochloride	Comedical s.r.l.	20 μg/mL
Methoxyacetyl norfentanyl hydrochloride	Comedical s.r.l.	50 μg/mL
N-piperidinyl etonitazene	Comedical s.r.l.	10 μg/mL
N-pyrrolidino etonitazene	Comedical s.r.l.	10 μg/mL
Para-fluoro-furanylfentanyl hydrochloride	Comedical s.r.l.	20 μg/mL
Protonitazene hydrochloride	Comedical s.r.l.	10 μg/mL
Trans-3-methyl norfentanyl	Comedical s.r.l.	50 μg/mL
β’-phenyl fentanyl	Comedical s.r.l.	50 μg/mL
β-hydroxy fentanyl hydrochloride	Comedical s.r.l.	50 μg/mL
β-hydroxy thiofentanyl hydrochloride	Comedical s.r.l.	50 μg/mL
2-methyl AP-237	Comedical s.r.l.	20 μg/mL
2′-fluoro, ortho-fluoro (±)-cis-3-methyn fentanyl	Comedical s.r.l.	10 μg/mL
Tryptamines		
5-methoxy-N,N-diallyltryptamine	Comedical s.r.l.	100 μg/mL
5-methoxy-N-methyl-N-isopropyltryptamine	Comedical s.r.l.	50 μg/mL
5-methoxy-α-methyltryptamine	Comedical s.r.l.	50 μg/mL

**Table 2:** Validation results obtained on spiked OF samples prepared at a 1:2 dilution ratio.

Substances	Interferences		LOI	Carryover
	Matrix	Inter-Analytes		
WG1				
4-FMC metabolite	N.O.	N.O.	100 ng/mL	N.O.
Methoxyacetyl norfentanyl	N.O.	N.O.	5 ng/mL	N.O.
2-CMC	N.O.	N.O.	10 ng/mL	N.O.
2-MMC	N.O.	N.O.	1 ng/mL	N.O.
3-CMC	N.O.	N.O.	5 ng/mL	N.O.
3-MMC	N.O.	N.O.	1 ng/mL	N.O.
4-CMC	N.O.	N.O.	10 ng/mL	N.O.
5-methoxy AMT	N.O.	N.O.	5 ng/mL	N.O.
2-FDCK	N.O.	N.O.	1 ng/mL	N.O.
4-MEC metabolite	N.O.	N.O.	5 ng/mL	N.O.
4-BMC	N.O.	N.O.	5 ng/mL	N.O.
Metodesnitazene	N.O.	N.O.	5 ng/mL	N.O.
WG2				
Deschloro-N-ethyl ketamine	N.O.	N.O	1 ng/mL	N.O.
6-MAPB	N.O.	N.O	5 ng/mL	N.O.
2-methyl-α-PPP	N.O.	N.O	10 ng/mL	N.O.
β-Pentedrone	N.O.	N.O	10 ng/mL	N.O.
5-methoxy MiPT	N.O.	N.O	1 ng/mL	N.O.
Furanyl fentanyl	N.O.	N.O	5 ng/mL	N.O.
Trans 3-methyl norfentanyl	N.O.	N.O	1 ng/mL	N.O.
Cis 3-methyl norfentanyl	N.O.	N.O	1 ng/mL	N.O.
Etodesnitazene	N.O.	N.O	1 ng/mL	N.O.
2C-B	N.O.	N.O	20 ng/mL	N.O.
N-ethyl pentylone	N.O.	N.O	5 ng/mL	N.O.
4-EEC	N.O.	N.O	10 ng/mL	N.O.
Butiryl norfentanyl	N.O.	N.O	10 ng/mL	N.O.
WG3				
3-F α-PVP	N.O.	N.O	5 ng/mL	N.O.
AP-237	N.O.	N.O	5 ng/mL	N.O.
3-HO-PCP	N.O.	N.O	1 ng/mL	N.O.
5-methoxy DALT	N.O.	N.O	10 ng/mL	N.O.
AP-238	N.O.	N.O	20 ng/mL	N.O.
2-methyl AP-237	N.O.	N.O	1 ng/mL	N.O.
Methoxyacetyl fentanyl	N.O.	N.O	1 ng/mL	N.O.
α-PiHP	N.O.	N.O	1 ng/mL	N.O.
β hydroxy thiofentanyl	N.O.	N.O	1 ng/mL	N.O.
α-PHP	N.O.	N.O	1 ng/mL	N.O.
Acetyl fentanyl	N.O.	N.O	1 ng/mL	N.O.
3-metossi-PCE	N.O.	N.O	5 ng/mL	N.O.
4-Cl-α-PVP	N.O.	N.O	1 ng/mL	N.O.
WG4				
4-F-α-PHP	N.O.	N.O	5 ng/mL	N.O.
MDPiHP	N.O.	N.O	1 ng/mL	N.O.
Etileneossinitazene	N.O.	N.O	1 ng/mL	N.O.
β hydroxy fentanyl	N.O.	N.O	1 ng/mL	N.O.
α-PHP metabolite	N.O.	N.O	5 ng/mL	N.O.
N-cyclohexyl butylone	N.O.	N.O	1 ng/mL	N.O.
N-pyrrolidin etonitazene	N.O.	N.O	1 ng/mL	N.O.
N-ethyl eptedrone	N.O.	N.O	1 ng/mL	N.O.
Brorphine	N.O.	N.O	1 ng/mL	N.O.
MPHP	N.O.	N.O	5 ng/mL	N.O.
1-Naphyrone	N.O.	N.O	1 ng/mL	N.O.
N-piperidinyl etonitazene	N.O.	N.O	20 ng/mL	N.O.
WG5				
Furanyl fentanyl	N.O.	N.O	1 ng/mL	N.O.
Pravadoline	N.O.	N.O	1 ng/mL	N.O.
Cyclopropyl fentanyl	N.O.	N.O	1 ng/mL	N.O.
p-F-furanyl fentanyl	N.O.	N.O	1 ng/mL	N.O.
1CP-LSD	N.O.	N.O	1 ng/mL	N.O.
Butiryl fentanyl	N.O.	N.O	1 ng/mL	N.O.
AM2233	N.O.	N.O	5 ng/mL	N.O.
3,4-Pr-PipVP	N.O.	N.O	1 ng/mL	N.O.
2′-F, o-F (±)-cis-3-metyl fentanyl	N.O.	N.O	1 ng/mL	N.O.
Protonitazene	N.O.	N.O	5 ng/mL	N.O.
Phenyl fentanyl	N.O.	N.O	1 ng/mL	N.O.
ADB-5Br-INACA	N.O.	N.O	100 ng/mL	N.O.
Butonitazene	N.O.	N.O	5 ng/mL	N.O.
Fluetizolam	N.O.	N.O	5 ng/mL	N.O.
WG6				
β-phenyl fentanyl	N.O.	N.O	1 ng/mL	N.O.
5-fluoro-APP-PICA	N.O.	N.O	5 ng/mL	N.O.
5-fluoro-MDMB-7-PAICA	N.O.	N.O	5 ng/mL	N.O.
ADB-BUTINACA	N.O.	N.O	20 ng/mL	N.O.
APP FUBINACA	N.O.	N.O	5 ng/mL	N.O.
5-fluoro-CUMYL-P7AICA	N.O.	N.O	5 ng/mL	N.O.
ADB-4en-PINACA	N.O.	N.O	5 ng/mL	N.O.
MMB2201	N.O.	N.O	5 ng/mL	N.O.
4-fluoro-MDMB-BUTICA	N.O.	N.O	5 ng/mL	N.O.
4-fluoro-MDMB-BUTINACA	N.O.	N.O	5 ng/mL	N.O.
BZO-4en-POXIZID	N.O.	N.O	5 ng/mL	N.O.
WG7				
5-fluoro-CUMYL-PICA	N.O.	N.O	1 ng/mL	N.O.
5-fluoro-CUMYL Pegaclone	N.O.	N.O	1 ng/mL	N.O.
5-fluoro-NNEI 2′-napthyl isomer	N.O.	N.O	5 ng/mL	N.O.
MDMB-4en-PICA	N.O.	N.O	1 ng/mL	N.O.
5C-MDA-19	N.O.	N.O	5 ng/mL	N.O.
5-fluoro-CUMYL-PINACA	N.O.	N.O	1 ng/mL	N.O.
MDMB-4en-PINACA metabolite	N.O.	N.O	5 ng/mL	N.O.
MDMB-BUTINACA	N.O.	N.O	5 ng/mL	N.O.
JWH-016	N.O.	N.O	1 ng/mL	N.O.
5-Cl-THJ 018	N.O.	N.O	100 ng/mL	N.O.
EDMB-PINACA	N.O.	N.O	5 ng/mL	N.O.
CUMYL-CH-MeGACLONE	N.O.	N.O	1 ng/mL	N.O.
WG8				
CUMYL-NBMINACA	N.O.	N.O	5 ng/mL	N.O.
9-HHC	N.O.	N.O	100 ng/mL	N.O.
JWH-147	N.O.	N.O	100 ng/mL	N.O.

Abbreviation: 4-FMC metabolite: 4-fluoromethcathinone metabolite; 2-CMC: 2-chloromethcathinone; 2-MMC: 2-methylmethcathinone; 3-CMC: 3-chloromethcathinone; 3-MMC: 3-methylmethcathinone; 4-CMC: 4-chloromethcathinone; 5-methoxy AMT: 5-methoxy-α-methyltryptamine; 2-FDCK: 2-fluorodeschloroketamine; 4-MEC metabolite: 4-methylethcathinone metabolite; 4-BMC: 4-bromomethcathinone; 2-methyl-α-PPP: 2-methyl-α-pyrrolidinopropiophenone; 5-methoxy MiPT: 5-methoxy-N-methyl-N-isopropyltryptamine; 4-EEC: 4-ethylethcathinone; 3-F α-PVP: 3-fluoro-α-pyrrolidinovalerophenone; 3-HO-PCP: 3-hydroxyphencyclidine; 5-methoxy DALT: 5-methoxy-N,N-diallyltryptamine; α-PiHP: α-pyrrolidinoisohexanophenone; α-PHP: α-pyrrolidinohexanophenone; 3-metossi-PCE: 3-methoxieticyclidine; 4-Cl-α-PVP: 4-chloro-α-pyrrolidinovalerophenone; 4-F-α-PHP: 4-fluoro-α-pyrrolidinohexanophenone; MDPiHP: 3,4-methylenedioxy-α-pyrrolidinohexanophenone; MPHP: 4-methyl-α-pyrrolidinohexanophenone; N.O: not observed; 3,4-Pr-PipVP: 3,4-trimethylene-α-Piperidinovalerophenone.

**Table 3:** Real OF samples analyzed, actual substances contained and analytes detected by analysis with the LC-HRMS/MS method.

Sample	Analyte Present	Analyte Detected	Results
OF1	HHC	N.A.	Negative
OF2	HHC	N.A.	Negative
OF3	α-PiHP	α-PiHP	Positive
OF4	3-CMC	3-CMC	Positive
OF5	3-CMC	3-CMC	Positive
OF6	Negativo	N.A.	Negative
OF7	α-PiHP	α-PiHP	Positive
OF8	α-PiHP	α-PiHP	Positive
OF9	carfentanyl	N.A.	Negative
OF10	Negative	N.A.	Negative
OF11	4-Cl-α-PVP	4-Cl-α-PVP	Positive
OF12	Negative	N.A.	Negative
OF13	Etodesnitazene	Etodesnitazene	Positive
OF14	Negative	N.A.	Negative
OF15	a-PHP	N.A.	False negative
OF16	Negative	N.A.	Negative
OF17	Negative	N.A.	Negative
OF18	3-CMC 2′-F-o-F-cis-3-methyl fentanyl	2′-F-o-F-cis-3-methyl fentanyl	Partially Positive
OF19	Iso-butirryl fentanyl Furanyl fentanyl	3-MMC; APP FUBINACA	False Positive
OF20	Negative	N.A.	Negative
OF21	CUMYL NBMINACA	CUMYL NBMINACA	Positive

## Data Availability

The original contributions presented in this study are included in the article/Supplementary Materials. Further inquiries can be directed to the corresponding author.

## Supplementary Materials

The following supporting information can be downloaded at: https://www.mdpi.com/article/10.3390/biology15080616/s1, Figure S1: WG1. Chromatograms (A) and fragmentation spectra (B) of the substances identified and confirmed by the UHPLC-HRMS/MS method. (a) 4-FMC metabolite, (b) methoxyacetyl norfentanyl, (c) 2-CMC, (d) 2-MMC, (e) 3-CMC, (f) 3-MMC, (g) 4-CMC, (h) 5-methoxy AMT, (i) 2-FDCK, (l) 4-MEC metabolite, (m) 4-BMC, (n) methodesnitazene; Figure S2: WG2. Chromatograms (A) and fragmentation spectra (B) of the substances identified and confirmed by the UHPLC-HRMS/MS method. (a) deschloro N ethyl ketamine, (b) 6-MAPB, (c) 2-methyl-α-PPP, (d) β-Pentedrone, (e) 5-methoxy MiPT, (f) furanyl norfentanyl, (g) trans 3-methyl norfentanyl, (h) cis 3-methyl norfentanyl, (i) etodesnitazene, (l) 2C-B, (m) N-ethyl pentylone, (n) 4-EEC, (o) butyryl norfentanyl; Figure S3: WG3. Chromatograms (A) and fragmentation spectra (B) of the substances identified and confirmed by the UHPLC-HRMS/MS method. (a) 3-F α-PVP, (b) AP-237, (c) 3-HO-PCP, (d) 5-methoxy DALT, (e) AP-238, (f) 2-methyl AP-237, (g) methoxyacetyl fentanyl, (h) α-PiHP, (i) β hydroxythiofentanyl, (l) α-PHP, (m) acetyl fentanyl, (n) 3-methoxy-PCE, (o) 4-Cl-α-PVP; Figure S4: WG4. Chromatograms (A) and fragmentation spectra (B) of the substances identified and confirmed by the UHPLC-HRMS/MS method. (a) 4-f α PHP, (b) MDPiHP, (c) ethyleneoxynitazene, (d) β hydroxyfentanyl, (e) α-PHP metabolite, (f) N-cyclohexyl butylone, (g) N-pyrrolidino etonitazene, (h) N-ethyl heptedrone, (i) brorphine, (l) MPHP, (m) 1-naphyrone, (n) N-piperidinyl etonitazene; Figure S5: WG5. Chromatograms (A) and fragmentation spectra (B) of the substances identified and confirmed by the UHPLC-HRMS/MS method. (a) furanyl fentanyl, (b) pravadoline, (c) cyclopropyl fentanyl, (d) p-F-furanyl fentanyl, (e) 1CP-LSD, (f) butyryl fentanyl, (g) AM2233, (h) 3,4-Pr-PipVP, (i) 2′-F, o-F (±)-cis-3-methyl fentanyl, (l) protonitazene, (m) phenyl fentanyl, (n) ADB-5Br-INACA, (o) butonitazene, (p) fluetizolam; Figure S6: WG6. Chromatograms (A) and fragmentation spectra (B) of the substances identified and confirmed by the UHPLC-HRMS/MS method. (a) β-phenylfentanyl, (b) 5-fluoro-APP-PICA, (c) 5-fluoro-MDMB-7-PAICA, (d) ADB-BUTINACA, (e) APP FU-BINACA, (f) 5-fluoro-CUMYL-P7AICA, (g) ADB-4en-PINACA, (h) MMB2201, (i) 4-Fluoro-MDMB-BUTICA, (l) 4-fluoro MDMB-BUTINACA, (m) BZO-4en-POXIZID; Figure S7: WG7. Chromatograms (A) and fragmentation spectra (B) of the substances identified and confirmed by the UHPLC-HRMS/MS method. (a) 5-fluoro-CUMYL-PICA, (b) 5-fluoro-CUMYL Pegaclone, (c) 5-fluoro NNEI 2′-naptyl isomer, (d) MDMB-4en-PINACA, (e) 5C-MDA-19, (f) 5-fluoro-CUMYL-PINACA, (g) MDMB-4en-PINACA metabolite, (h) MDMB-BUTINACA, (i) = JWH-016, (l) 5-chloro THJ 018, (m) EDMB-PINACA, (n) CUMYL-CH-MeGACLONE; Figure S8: WG8 group. Chromatograms (A) and fragmentation spectra (B) of the substances iden-tified and confirmed by the UHPLC-HRMS/MS method. (a) CUMYL-NBMINACA, (b) 9-HHC, (c) JWH–147; Table S1: Precursor ions, main identifying fragments and retention times of the analyzed standards.

## Abbreviations

The following abbreviations are used in this manuscript:

UNODC	United Nations Office on Drugs and Crime
NPS	New Psychoactive Substances
SCRAs	Synthetic cannabinoids
SC	Synthetic cathinones
OF	Oral Fluid
HRMS	High resolution mass spectrometry
UHPLC-HRMS/MS	Ultra-high-performance liquid chromatography coupled with a quadrupole-Orbitrap hybrid mass spectrometer
LC-MS	Liquid chromatography-mass spectrometry
MeOH	Methanol
ACN	Acetonitrile
MPA	Mobile phase A
MPB	Mobile phase B
WG	Working Group
Full MS	Full Mass
ddMS^2^	Data-dependent MS/MS
AGC	Automatic gain control
IT	Injection time
NCE	Normalized collision energies
LOI	Limit of Identification
LOD	Limit of Detection
2C-B	4-bromo-2,5-dimethoxyphenylethylamine
3,4-Pr-PipVP	3,4-trimethylene-α-Piperidinovalerophenone
AMT	α-methyltryptamine
BMC	Bromomethcathinone
Br	Bromine
Cl	Chlorine
CMC	Chloromethcathinone
DALT	Diallyltryptamine
EEC	Ethylethcathinone
F	Fluorine
FDCK	Fluorodeschloroketamine
FMC	Fluoromethcathinone
HHC	Hexahydrocannabinol
HHCH	Hexahydrocannabiesol
HHCP	Hexahydrocannabiphorol
MDPiHP	3,4-methylenedioxypyrrolidinoisohexanophenone
MeO	Methoxy
MiPT	N-methyl-N-isopropyltryptamine
MMC	Methylmethcathinone
MPHP	4-methyl-α-pyrrolidinohexanophenone
o	Acetate
OH	Hydroxy
PCE	Ethicyclidine
PCP	Phencyclidine
PHP	Pyrrolidinohexanophenone
PiHP	Pyrrolidinoisohexanophenone
PPP	Pyrrolidinopropiophenone
PVP	Pyrrolidinovalerophenone
