# Supplementary material for: A Comprehensive HPLC-HRMS/MS Targeted Screening Method to Detect 90 New Psychoactive Substances in Oral Fluid Samples
Source: Biology (Basel). 2026 Apr 13;15(8):616. doi: 10.3390/biology15080616 (PMC13113579; doi:10.3390/biology15080616)
Supplement: Supplementary file 1 [file biology-15-00616-s001.zip › biology-4214504-supplementary.pdf]

Supplementary Material

Figure S1. WG1. Chromatograms (A) and fragmentation spectra (B) of the substances identified and confirmed by the UHPLC-HRMS/MS method. (a) 4-FMC metabolite, (b) methoxyacetyl norfentanyl, (c) 2-CMC, (d) 2-MMC, (e) 3-CMC, (f) 3-MMC, (g) 4-CMC, (h) 5-methoxy AMT, (i) 2-FDCK, (l) 4-MEC metabolite, (m) 4-BMC, (n) methodesnitazene.

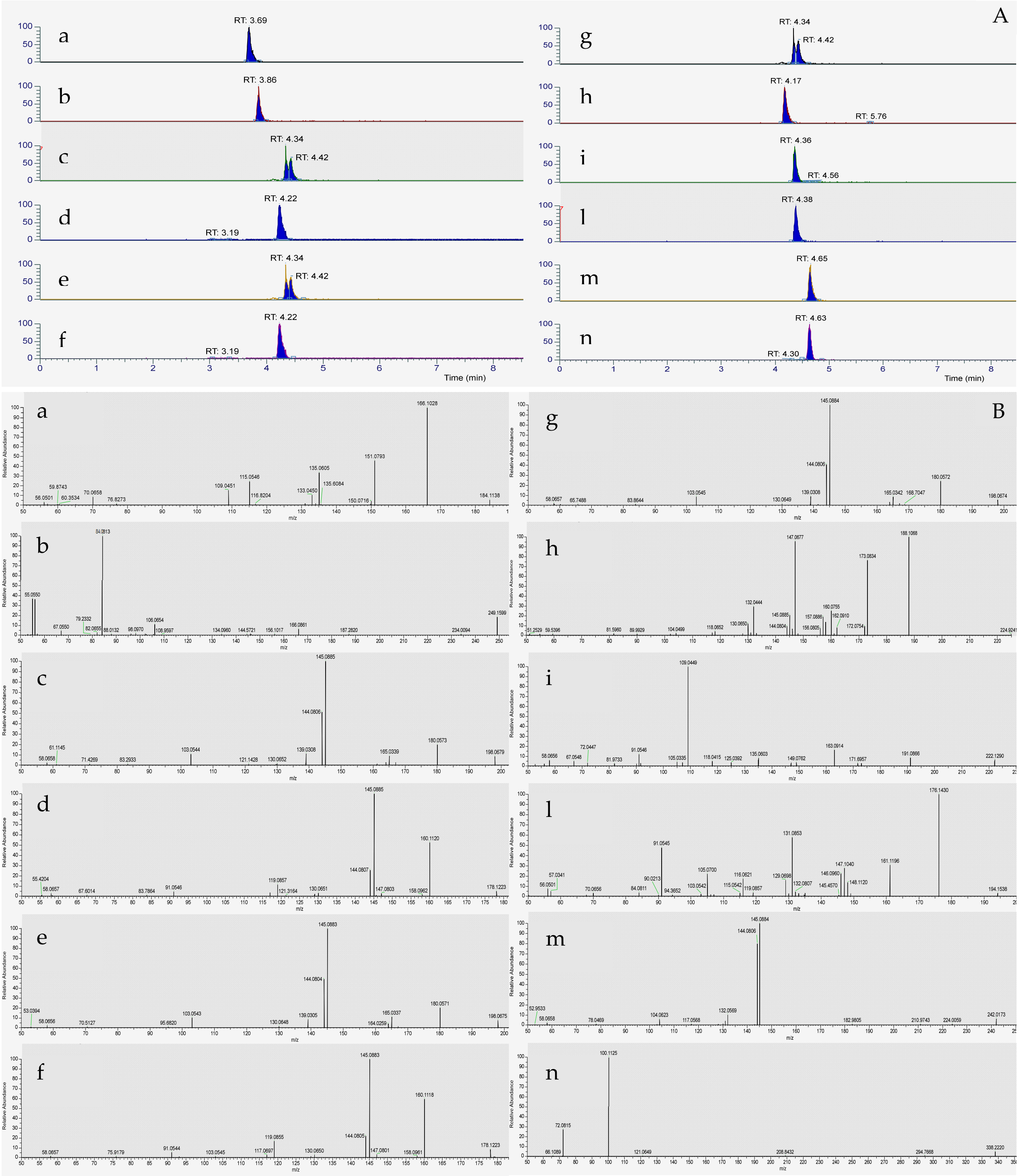

Figure S2. WG2. Chromatograms (A) and fragmentation spectra (B) of the substances identified and confirmed by the UHPLC-HRMS/MS method. (a) deschloro N ethyl ketamine, (b) 6-MAPB, (c) 2-methyl-α-PPP, (d) β-Pentedrone, (e) 5-methoxy MiPT, (f) furanyl norfentanyl, (g) trans 3-methyl norfentanyl, (h) cis 3-methyl norfentanyl, (i) etodesnitazene, (l) 2C-B, (m) N-ethyl pentylone, (n) 4 – EEC, (o) butyryl norfentanyl.

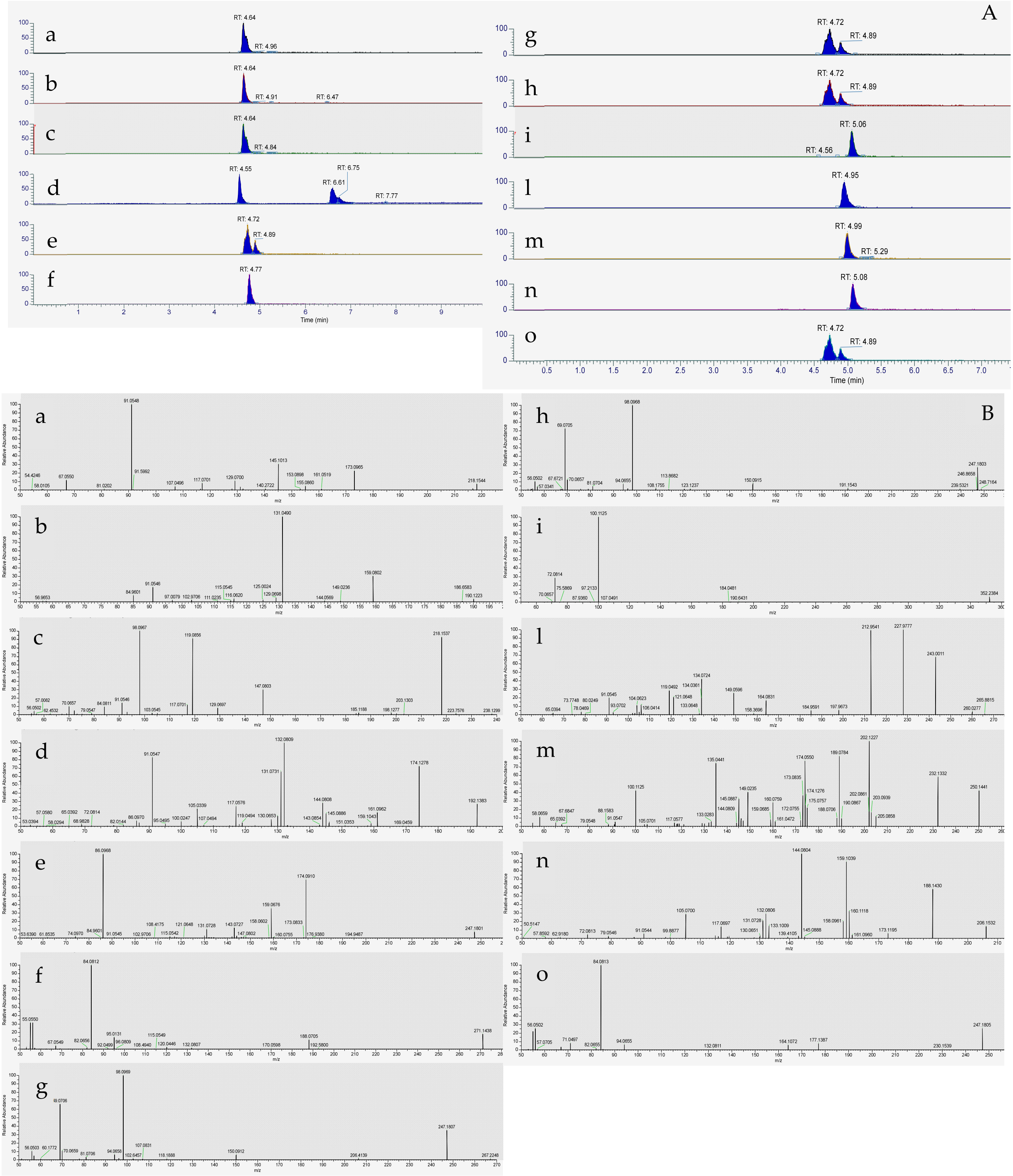

Figure S3. WG3. Chromatograms (A) and fragmentation spectra (B) of the substances identified and confirmed by the UHPLC-HRMS/MS method. (a) 3-F α-PVP, (b) AP-237, (c) 3-HO-PCP, (d) 5-methoxy DALT, (e) AP-238, (f) 2-methyl AP-237, (g) methoxyacetyl fentanyl, (h) α – PiHP, (i) β hydroxythiofentanyl, (l) α-PHP, (m) acetyl fentanyl, (n) 3-methoxy-PCE, (o) 4-Cl-α-PVP.

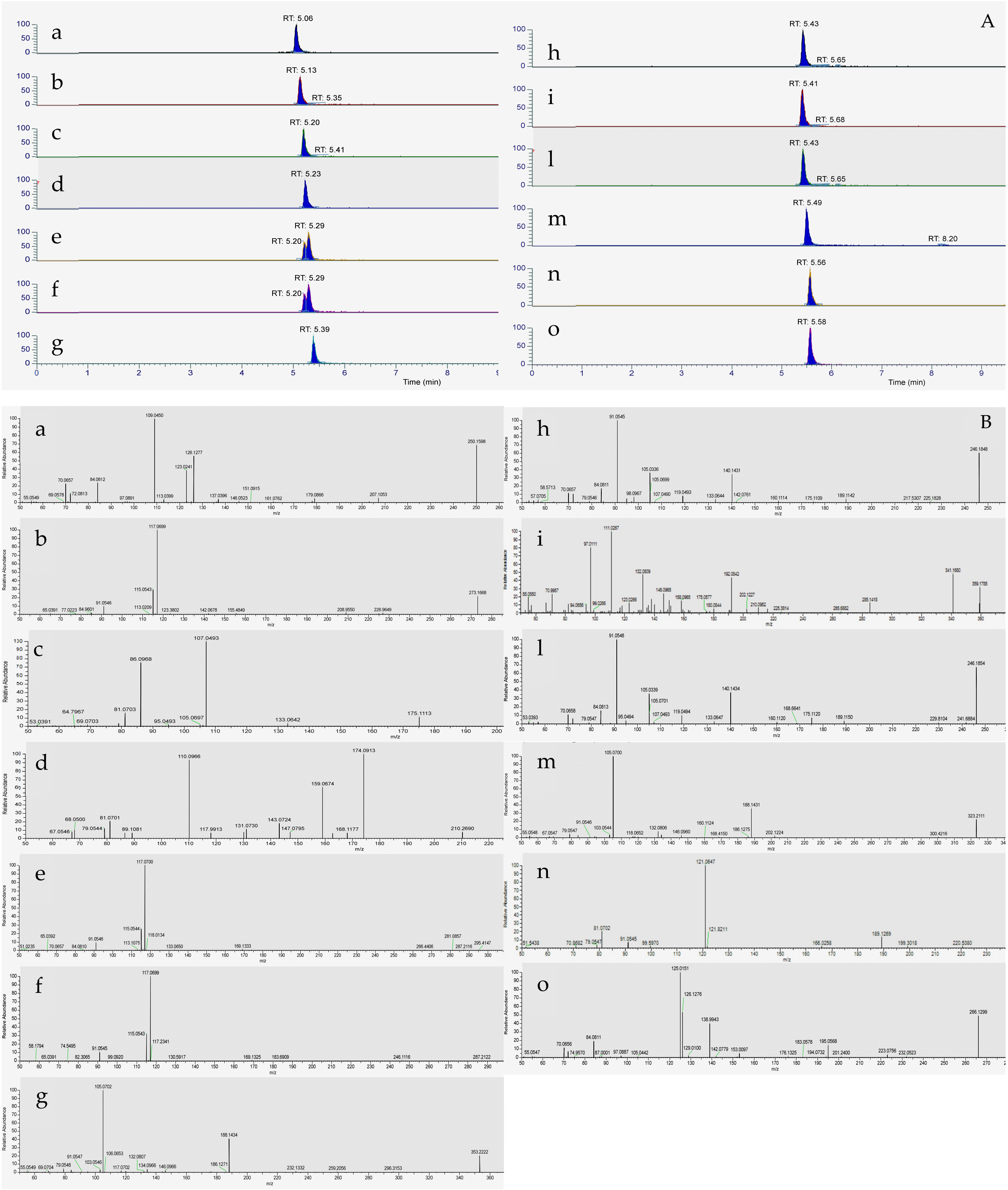

Figure S4. WG4. Chromatograms (A) and fragmentation spectra (B) of the substances identified and confirmed by the UHPLC-HRMS/MS method. (a) 4-f α PHP, (b) MDPiHP, (c) ethyleneoxynitazene, (d) β hydroxyfentanyl, (e) α-PHP metabolite, (f) N-cyclohexyl butylone, (g) N-pyrrolidino etonitazene, (h) N-ethyl heptedrone, (i) brorphine, (l) MPHP, (m) 1-naphyrone, (n) N-piperidinyl etonitazene.

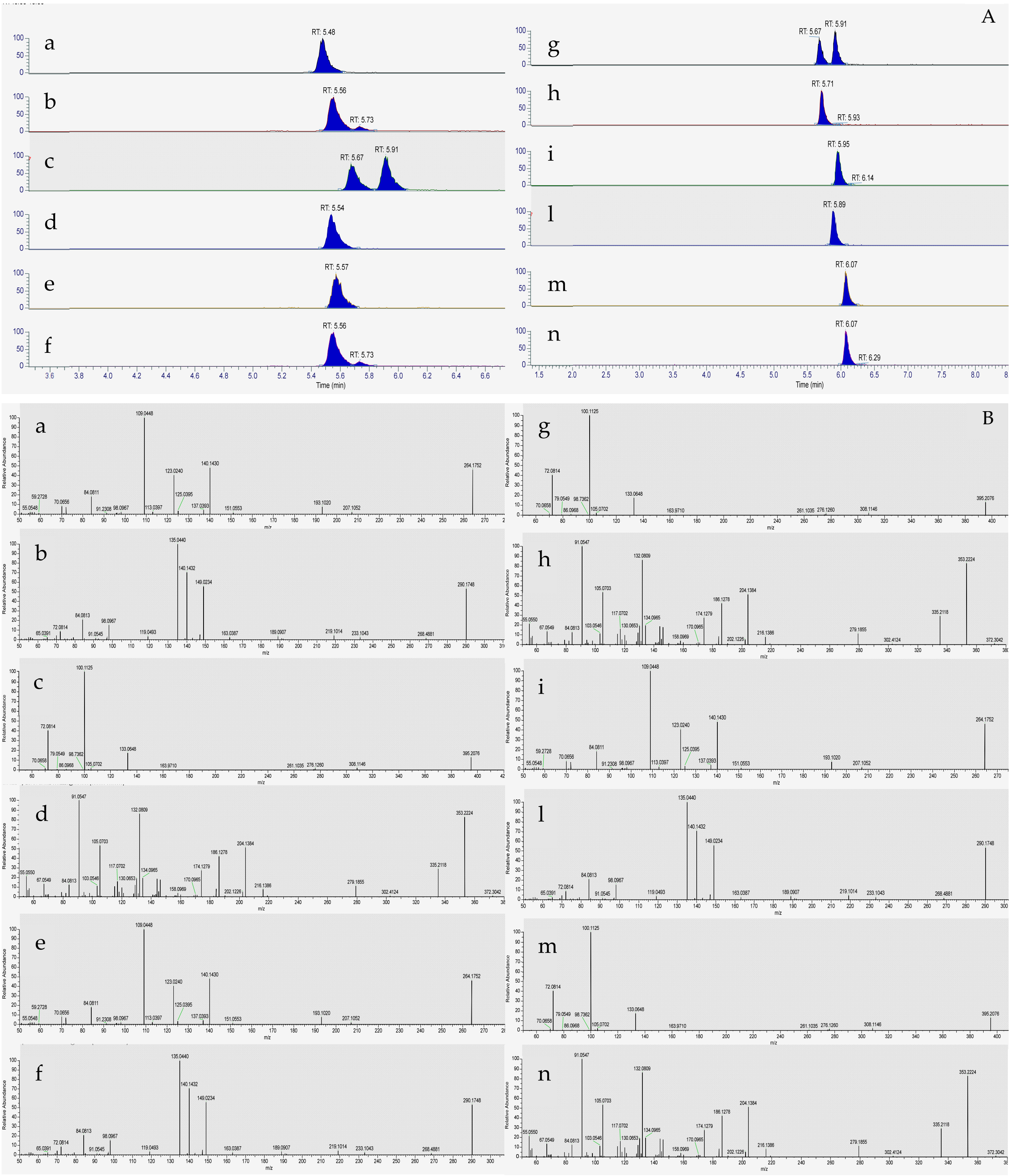

Figure S5. WG5. Chromatograms (A) and fragmentation spectra (B) of the substances identified and confirmed by the UHPLC-HRMS/MS method. (a) furanyl fentanyl, (b) pravadoline, (c) cyclopropyl fentanyl, (d) p-F-furanyl fentanyl, (e) 1CP-LSD, (f) butyryl fentanyl, (g) AM2233, (h) 3,4-Pr-PipVP, (i) 2'-F, o-F (±)-cis-3-methyl fentanyl, (l) protonitazene, (m) phenyl fentanyl, (n) ADB - 5Br – INACA, (o) butonitazene, (p) fluetizolam.

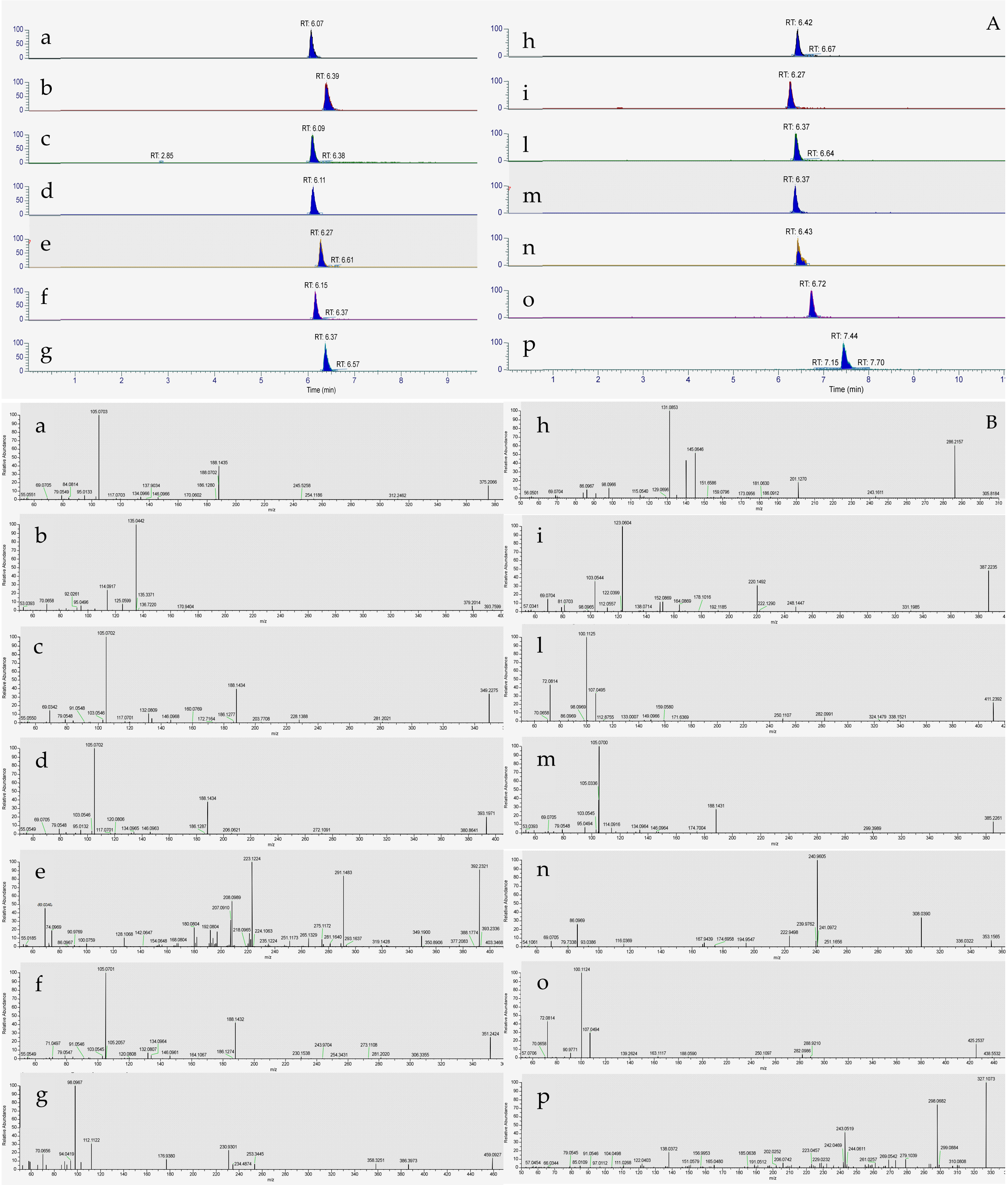

Figure S6. WG6. Chromatograms (A) and fragmentation spectra (B) of the substances identified and confirmed by the UHPLC-HRMS/MS method. (a) β-phenylfentanyl, (b) 5-fluoro-APP-PICA, (c) 5-fluoro-MDMB-7-PAICA, (d) ADB-BUTINACA, (e) APP FU-BINACA, (f) 5-fluoro-CUMYL-P7AICA, (g) ADB-4en-PINACA, (h) MMB2201, (i) 4-Fluoro- MDMB-BUTICA, (l) 4-fluoro MDMB-BUTINACA, (m) BZO-4en-POXIZID.

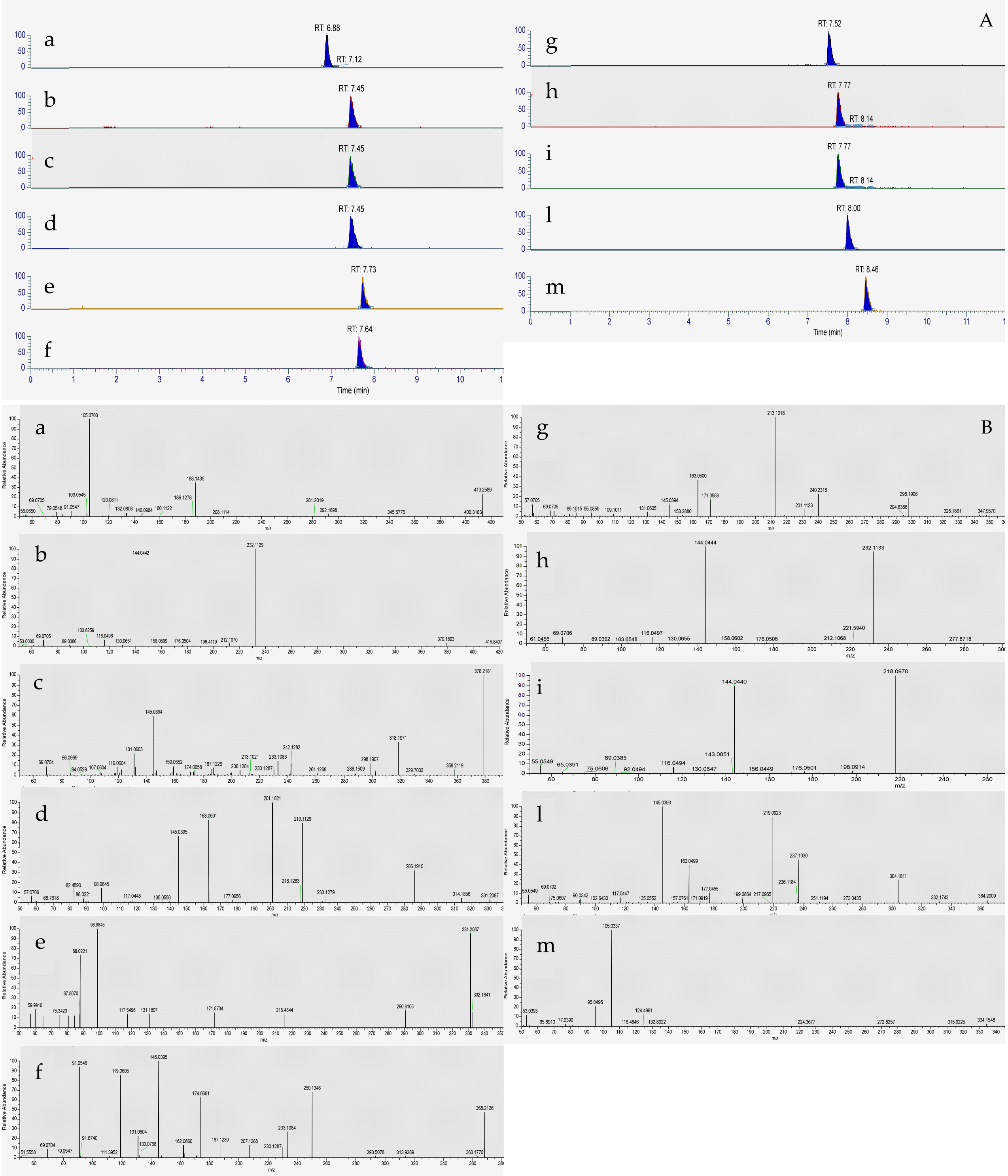

Figure S7. WG7. Chromatograms (A) and fragmentation spectra (B) of the substances identified and confirmed by the UHPLC-HRMS/MS method. (a) 5-fluoro-CUMYL-PICA, (b) 5-fluoro-CUMYL Pegaclone, (c) 5-fluoro NNEI 2'-naptyl isomer, (d) MDMB-4en-PINACA, (e) 5C-MDA-19, (f) 5-fluoro-CUMYL-PINACA, (g) MDMB-4en-PINACA metabolite, (h) MDMB-BUTINACA,(i) = JWH-016, (l) 5-chloro THJ 018, (m) EDMB-PINACA, (n) CUMYL-CH-MeGACLONE.

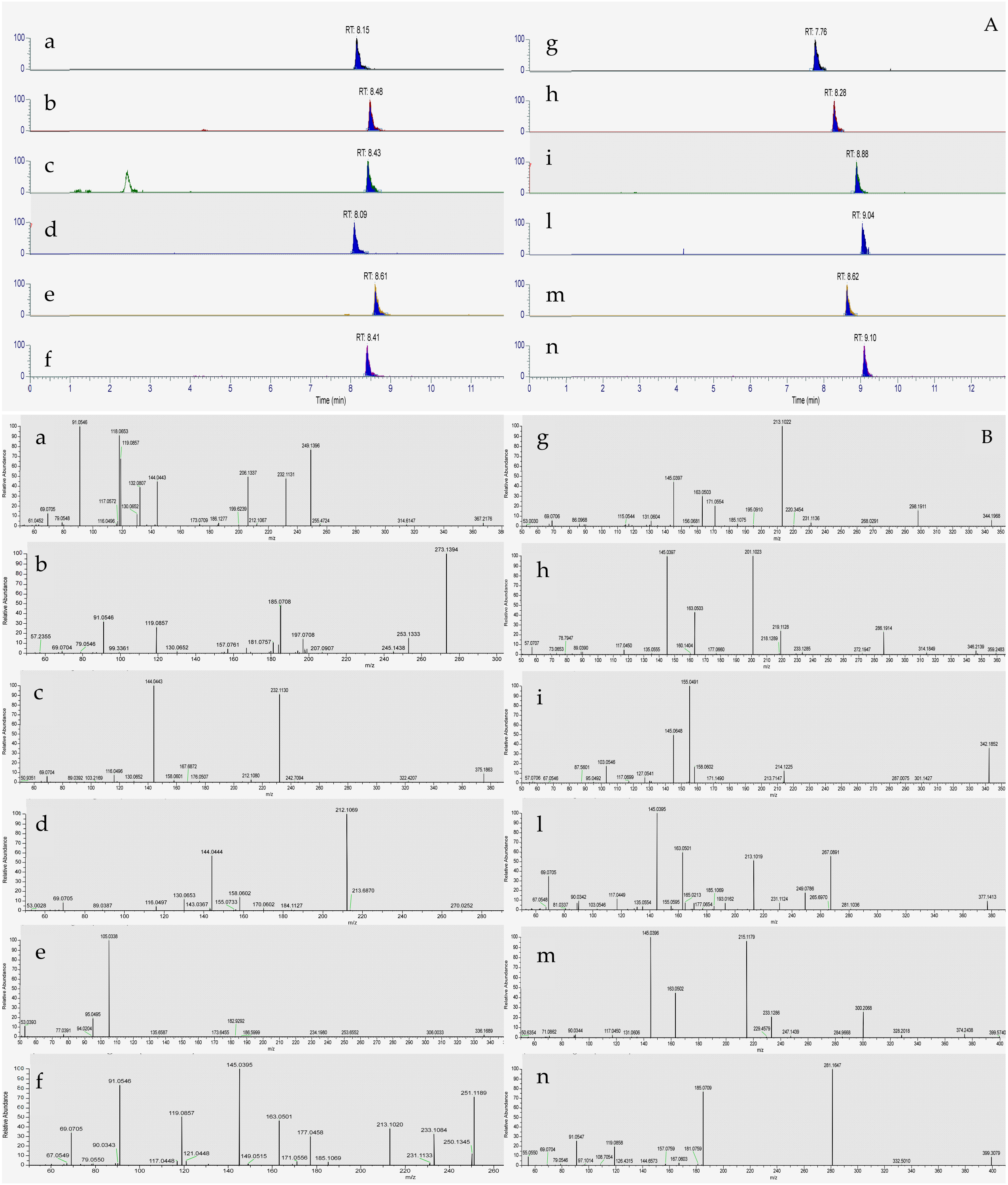

Figure S8. WG8 group. Chromatograms (A) and fragmentation spectra (B) of the substances iden-tified and confirmed by the UHPLC-HRMS/MS method. (a) CUMYL-NBMINACA, (b) 9()-HHC, (c) JWH–147.

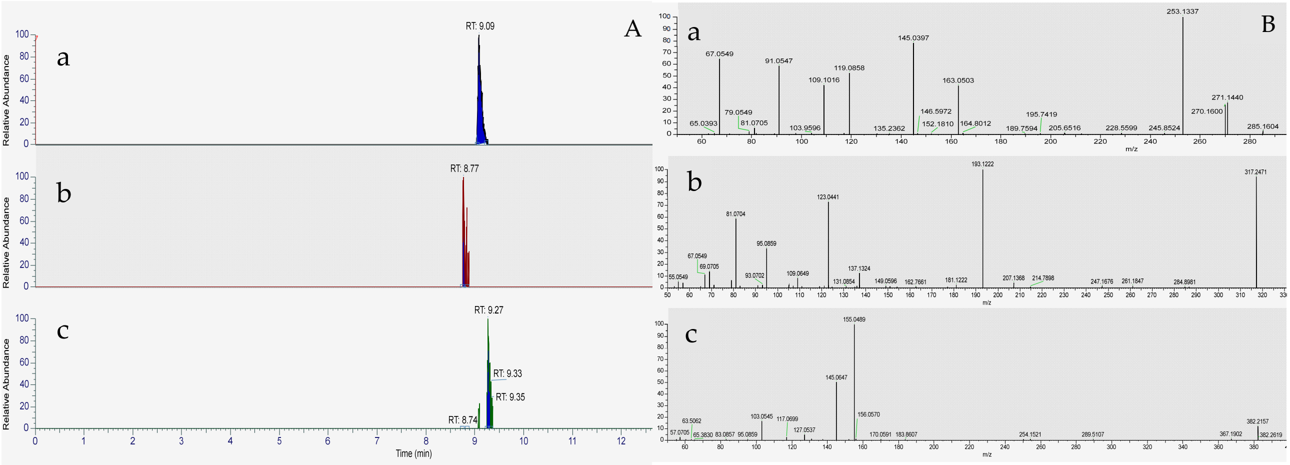

Table S1. Precursor ions, main identifying fragments and retention times of the analyzed standards.

Name	Class	Formula	RT	[M+ H] m/z	m/z fragments
1- Naphyrone	Synthetic cathinones	C19H23NO	6.07	282.1850	126.1278<br>141.0689<br>155.0491<br>169.0650<br>211.1116<br>282.1850
2C-B	Phenethylamines	C10H14BrNO2	4.94	260.0276	91.6768<br>212.9546<br>227.9780<br>243.0014
2'-F. o-F(±)-cis-3-methylfentanyl	Synthetic opioids	C23H28F2N2O	6.26	387.2237	69.0705<br>103.0545<br>123.0606<br>152.0807<br>220.1495<br>331.1978

					387.2237
2-FDCK	Arylcyclohexylamines	C13H16FNO	4.35	222.1288	67.0549<br>109.0450<br>147.0640<br>163.0917<br>191.0866<br>204.1183
2-methyl AP-237	Synthetic opioids	C18H26N2O	5.20	287.2115	115.0544<br>117.0700
2-methyl-α-PPP	Synthetic cathinones	C14H19NO	4.64	218.1538	98.0968<br>119.0857<br>129.0700<br>147.0803<br>218.1538
3-CMC	Synthetic cathinones	C10H12ClNO	4.34	198.068	103.0543<br>139.0305<br>144.0804<br>145.0883<br>165.0337<br>180.0571
3-MMC	Synthetic cathinones	C11H15NO	4.22	178.1222	119.0855<br>144.0805<br>145.0883<br>160.1118
3-methoxy-PCE	Arylcyclohexylamines	C15H23NO	5.55	234.1846	81.0702<br>91.0545<br>121.0647<br>166.0258<br>189.1269<br>220.5380
3.4-Pr-PipVP	Synthetic cathinones	C19H27NO	6.41	286.2158	98.0966

					115.0544<br>131.0853<br>140.1431<br>145.0647<br>201.1269<br>243.1615
3-F-α-PVP	Synthetic cathinones	C15H20FNO	5.04	250.16	70.0657<br>84.0812<br>109.0449<br>126.1277<br>179.0864<br>207.1051
3-HO-PCP	Arylcyclohexylamines	C17H25NO	4.95	260.2002	86.0968<br>107.0493<br>133.0642<br>175.1113
4-BMC	Synthetic cathinones	C10H12BrNO	4.62	242.0172	104.0623<br>132.0569<br>144.0806<br>145.0884<br>182.9805
4-CMC	Synthetic cathinones	C10H12ClNO	4.34	198.068	103.0545<br>139.0308<br>144.0806<br>145.0884<br>165.0342<br>180.0572
4-EEC	Synthetic cathinones	C13H19NO	5.08	206.15394	105.07<br>117.0697<br>132.0806<br>144.0804

					159.1039<br>188.143
4-Cl-α-PVP	Synthetic cathinones	C15H20ClNO	5.56	266.1299	84.0811<br>125.0151<br>126.1276<br>138.9943<br>195.0568
4-F-α-PHP	Synthetic cathinones	C16H22FNO	5.47	264.1753	84.0811<br>109.0448<br>123.0240<br>140.1430<br>193.1020
4-F-MDMB-BUTINACA	Synthetic cannabinoids	C19H26FN3O3	8.00	364.202	145.0393<br>163.0499<br>219.0923<br>237.1030<br>304.1811
4-F-MDMB-BUTICA	Synthetic cannabinoids	C20H27FN2O3	7.82	363.2067	116.0494<br>144.0440<br>218.097
4-MEC metabolite	Synthetic cathinones	C12H19NO	3.34	194.1536	91.0545<br>105.0700<br>116.0621<br>129.0698<br>131.0853<br>146.0960<br>147.1040<br>161.1196<br>176.1430
MPHP	Synthetic cathinones	C17H25NO	5.88	260.2007	84.0812<br>105.0701

					119.0492
					133.0646
					140.1432
					189.1274
5-Cl-THJ 018	Synthetic cannabinoids	C23H21ClN2O	9.06	377.141	145.0395
					163.0501
					213.1019
					249.0786
					267.0891
5C-MDA-19	Synthetic cannabinoids	C20H21N3O2	8.64	336.1701	53.0393
					95.0495
					105.0338
5-F-NNEI 2'-napthyl isomer	Synthetic cannabinoids	C24H23FN2O	8.44	375.1861	116.0496
					144.0442
					232.1128
5-F-APP-PICA	Synthetic cannabinoids	C23H26FN3O2	7.45	396.2077	116.0496
					144.0442
					232.1129
5-F-CUMYL Pegaclone	Synthetic cannabinoids	C25H27FN2O	8.49	391.2175	91.0546
					119.0857
					185.0708
					197.0708
					253.1333
					273.1394
5-fluoro-CUMYL-P7AICA	Synthetic cannabinoids	C22H26FN3O	7.64	368.2126	91.0546
					119.0605
					131.0604
					145.0395
					162.0660
					174.0661
					187.1230

					207.1288<br>233.1084<br>250.1348
5-F-CUMYL-PICA	Synthetic cannabinoids	C23H27FN2O	8.17	367.2176	91.0546<br>118.0653<br>119.0857<br>132.0807<br>144.0443<br>206.1337<br>232.1131<br>249.1396
5-F-CUMYL-PINACA	Synthetic cannabinoids	C22H26FN3O	8.42	368.2125	69.0705<br>91.0546<br>119.0857<br>145.0395<br>163.0501<br>177.0458<br>213.1020<br>233.1084<br>251.1189
5-F-MDMB-7-PAICA	Synthetic cannabinoids	C20H28FN3O3	7.45	378.2181	119.0604<br>131.0603<br>145.0394<br>159.0552<br>233.1083<br>242.1282<br>298.1907<br>318.1971
5-methoxy AMT	Synthetic tryptamines	C12H16N2O	4.16	205.1335	130.0650<br>132.0444<br>145.0885

					147.0677<br>160.0755<br>173.0834<br>188.1068
5-methoxy DALT	Synthetic tryptamines	C17H22N2O	5.28	271.1804	79.0544<br>81.0701<br>110.0966<br>117.9913<br>131.0730<br>143.0724<br>159.0674<br>174.0913<br>210.2690
5-methoxy MiPT	Synthetic tryptamines	C15H22N2O	4.69	247.1801	86.0968<br>131.0728<br>143.0727<br>159.0676<br>174.0910
6-MAPB	Phenethylamines	C12H15NO	4.65	190.1223	84.9601<br>91.0546<br>129.0698<br>131.0490<br>131.0853<br>159.0802
9-HHC	Synthetic cannabinoids	C21H32O2	8.79	317.2647	81.0703<br>95.0858<br>109.0649<br>123.0440<br>137.0595<br>193.1219<br>207.1383

					317.2467
Acetyl fentanyl	Synthetic opioids	C21H26N2O	5.50	323.2111	103.0543<br>105.0700<br>132.0805<br>188.1429<br>202.1222<br>323.2111
ADB-4en-PINACA	Synthetic cannabinoids	C19H26N4O2	7.54	343.212	131.0605<br>145.0394<br>163.0500<br>171.0553<br>213.1018<br>231.1123<br>240.2318<br>298.1906
ADB-BUTINACA	Synthetic cannabinoids	C18H26N4O2	7.45	331.2125	98.9845<br>117.0448<br>145.0395<br>163.0501<br>177.0656<br>201.1021<br>219.1126<br>233.1279<br>286.1910<br>314.1856
AM2233	Synthetic cannabinoids	C22H23IN2O	6.39	459.0924	70.0658<br>94.0417<br>98.0968<br>112.1123<br>230.9294<br>362.0037

AP-237	Synthetic opioids	C17H24N2O	5.13	273.1957	91.0546<br>115.0543<br>117.0699
AP-238	Synthetic opioids	C18H26N2O	5.28	287.2214	91.0546<br>115.0544<br>117.0700
APP FUBINACA	Synthetic cannabinoids	C24H21FN4O2	7.74	417.1721	109.045<br>225.0823<br>253.0767<br>271.0875<br>372.1494
Brorphine	Synthetic opioids	C20H22BrN3O	5.95	400.1013	84.0812<br>104.0623<br>135.0552<br>173.0708<br>182.9802<br>218.1285
Butonitazene	Synthetic opioids	C24H32N4O3	6.72	425.2542	72.0814<br>100.1124<br>107.0494<br>282.0996
Butyryl fentanyl	Synthetic opioids	C23H30N2O	6.16	351.2425	103.0545<br>105.0701<br>132.0807<br>146.0961<br>188.1432<br>230.1538<br>351.2425
Butyryl norfentanyl	Synthetic opioids	C15H22N2O	4.89	247.1804	84.0813<br>94.0655<br>94.0665

					164.1062<br>177.1387<br>247.1804
BZO-4en-POXIZID	Synthetic cannabinoids	C20H19N3O2	8.46	334.1543	95.0495<br>105.0337<br>105.0440
Cis 3-methyl norfentanyl	Synthetic opioids	C15H22N2O	4.72	247.1803	69.0705<br>94.0655<br>98.0968<br>150.0915
CUMYL-CH-MeGACLONE	Synthetic cannabinoids	C27H30N2O	9.11	399.2429	91.0547<br>119.0858<br>185.0710<br>281.1648
CUMYL-NBMINACA	Synthetic cannabinoids	C25H29N3O	9.11	388.238	67.0549<br>91.0547<br>109.1016<br>119.0858<br>145.0397<br>163.0503<br>253.1337<br>270.1600<br>271.1440<br>285.0160
Cyclopropyl fentanyl	Synthetic opioids	C23H28N2O	6.09	349.2272	69.0342<br>103.0546<br>105.0702<br>132.0809<br>146.0968<br>188.1434<br>238.1388

					349.2275
Deschloro-N-ethyl-ketamine	Arylcyclohexylamines	C14H19NO	4.65	218.1538	67.055<br>91.0548<br>107.0496<br>117.0701<br>129.0700<br>145.1013<br>173.0965<br>218.1544
EDMB-PINACA	Synthetic cannabinoids	C21H31N3O3	8.64	374.2436	145.0396<br>163.0502<br>215.1179<br>233.1286<br>300.2068
Ethyleneoxynitazene	Synthetic opioids	C22H26N4O3	5.68	395.2075	72.0814<br>100.1125<br>133.0648<br>395.2076
Etodesnitazene	Synthetic opioids	C22H29N3O	5.07	352.238	72.0814<br>100.1125
4-FMC metabolite	Synthetic cathinones	C10H14FNO	3.69	184.1132	70.0658<br>109.0451<br>115.0546<br>115.0546<br>133.0450<br>135.0605<br>151.0793<br>166.1028<br>184.1138
Fluetizolam	Benzodiazepine	C17H15FN4S	7.44	327.1073	138.0372<br>206.0742

					243.0519<br>298.0682<br>327.1073
Furanyl norfentanyl	Synthetic opioids	C16H18N2O2	4.76	271.1438	56.0502<br>84.0812<br>95.0131<br>188.0705<br>271.1438
Furanyl fentanyl	Synthetic opioids	C24H26N2O2	6.07	375.2065	95.0133<br>103.0547<br>105.0703<br>188.1435<br>375.2066
JWH-016	Synthetic cannabinoids	C24H23NO	8.90	342.1852	103.0545<br>127.054<br>145.0647<br>155.0491<br>158.0600<br>214.1224
MDMB-4en-PICA	Synthetic cannabinoids	C21H28N2O3	8.11	357.2169	69.0705<br>116.0497<br>130.0653<br>144.0444<br>158.0602<br>212.1069
MDMB-4en-PINACA metabolite	Synthetic cannabinoids	C17H25N3O3	7.79	344.1968	131.0604<br>145.0397<br>163.0503<br>171.0554<br>213.1022<br>298.1911

					344.1968
MDMB-BUTINACA	Synthetic cannabinoids	C19H27N3O3	8.29	346.2122	57.0707<br>117.0450<br>145.0397<br>163.0503<br>201.1023<br>219.1128<br>286.1914
Methoxyacetyl fentanyl	Synthetic opioids	C22H28N2O2	5.38	353.2221	103.0546<br>105.0702<br>117.0702<br>134.0966<br>188.1434<br>353.2222
Methoxyacetyl norfentanyl	Synthetic opioids	C14H20N2O2	3.86	249.1596	55.055<br>84.0813<br>106.0654<br>166.0861
Metodesnitazene	Synthetic opioids	C21H27N3O	4.63	338.2224	72.0815<br>100.1125<br>338.222
MMB2201	Synthetic cannabinoids	C20H27FN2O3	7.77	363.2078	69.0706<br>116.0497<br>144.0444<br>232.1133
N-cyclohexyl butylone	Synthetic cathinones	C17H23NO3	5.73	290.1747	83.086<br>105.0704<br>117.0577<br>132.0808<br>133.0647<br>143.0231

					160.0757<br>161.0597<br>190.0862<br>191.0698<br>208.0968
N-ethyl heptedrone	Synthetic cathinones	C15H23NO	5.72	234.1853	91.0548<br>105.0339<br>107.0495<br>117.0577<br>118.0655<br>130.0653<br>145.0887<br>146.0965<br>158.0964
N-ethyl pentylone	Synthetic cathinones	C14H19NO3	5.00	250.1436	100.1125<br>135.0441<br>145.0887<br>149.0235<br>160.0759<br>173.0835<br>174.055<br>189.0784<br>202.1227<br>232.1332<br>250.1441
N-piperidinyl etonitazene	Synthetic opioids	C23H28N4O3	6.07	409.2232	69.0706<br>84.0814<br>107.0495<br>112.1124<br>135.0806
N-pyrrolidin etonitazene	Synthetic opioids	C22H26N4O3	5.90	395.2075	98.0967

					107.0493<br>135.0805
p-F-furanyl fentanyl	Synthetic opioids	C24H25FN2O2	6.10	393.1969	79.0548<br>95.0132<br>103.0546<br>105.0702<br>186.1287<br>188.1434<br>272.1091<br>393.1971
Phenyl fentanyl	Synthetic opioids	C26H28N2O	6.37	385.2271	105.0703<br>146.0964<br>188.1434
Pravadoline	Synthetic cannabinoids	C23H26N2O3	6.40	379.2016	70.0658<br>95.0496<br>105.0452<br>114.0917<br>125.0599<br>135.0442
Protonitazene	Synthetic opioids	C23H30N4O3	6.38	411.239	72.0814<br>100.1125<br>107.0495<br>282.0991<br>411.2392
Trans 3-methyl norfentanyl	Synthetic opioids	C15H22N2O	4.72	247.1805	69.0706<br>96.0658<br>98.0969<br>150.0912<br>247.1807
α-PiHP	Synthetic cathinones	C16H23NO	5.43	246.1853	84.0813<br>91.0547

					105.0339
					119.0494
					133.0649
					140.1434
					189.1149
					246.1853
α-PHP	Synthetic cathinones	C16H23NO	5.43	246.1852	84.0813
					91.0548
					105.0339
					119.0494
					133.0647
					140.1434
					175.1120
					189.1100
					246.1854
α-PHP metabolite	Synthetic cathinones	C16H25NO	5.57	248.2009	72.0815
					79.0549
					91.0548
					104.0625
					107.0496
					117.0702
					173.1199
					230.1906
					248.2010
β-hydroxy fentanyl	Synthetic opioids	C22H28N2O2	5.55	353.2223	91.0547
					105.0703
					117.0702
					130.0653
					132.0809
					134.0965
					144.0809

					146.0966<br>174.1279<br>186.1278<br>204.1384<br>279.1855<br>335.2218
β-hydroxy thiofentanyl	Synthetic opioids	C20H26N2O2S	5.41	359.1786	97.0111<br>111.0267<br>123.0266<br>132.0809<br>146.0965<br>158.0968<br>192.0842<br>285.1418<br>341.1680<br>359.1785
β-Pentedrone	Synthetic cathinones	C12H17NO	4.54	192.1383	91.0548<br>105.0339<br>117.0577<br>128.0196<br>130.0654<br>131.0732<br>132.0810<br>145.0888<br>161.0960<br>174.1279
β-phenyl fentanyl	Synthetic opioids	C28H32N2O	6.92	413.2585	91.0547<br>105.0703<br>132.0808<br>188.1435
2-CMC	Synthetic cathinones	C10H12ClNO	4.43	198.0678	103.0544

					139.0308<br>144.0806<br>145.0885<br>165.0339<br>180.0573<br>198.0679
2-MMC	Synthetic cathinones	C11H15NO	4.23	178.1225	91.0546<br>119.0857<br>130.0651<br>144.0807<br>145.0885<br>160.1120<br>178.1223
MDPiHP	Synthetic cathinones	C17H23NO3	5.55	290.1747	84.0813<br>98.0968<br>135.0440<br>140.1433<br>149.0233<br>219.1014
1CP-LSD	Synthetic cannabinoids	C24H29N3O2	6.26	392.2329	69.0341<br>74.097<br>90.977<br>128.1070<br>180.0806<br>192.0809<br>207.0913<br>208.0755<br>223.1227<br>291.1490<br>392.2329
JWH-147	Synthetic cannabinoids	C27H27NO	9.29	382.2161	103.0545

					117.0699<br>127.0537<br>131.0488<br>145.0647<br>155.0489<br>367.1902
ADB-5Br-INACA	Synthetic cannabinoids	C14H17BrN4O2	6.43	353.0603	86.0969<br>116.0369<br>194.9552<br>222.9497<br>240.9603<br>308.0388<br>336.0340
